# A real-world pharmacovigilance study of lorazepam based on the FDA adverse event reporting system database

**DOI:** 10.1038/s41598-025-05680-z

**Published:** 2025-06-24

**Authors:** Chunyue Fang, Xiaoyan Xu, Jianyi Li, Yuanyuan Zhong, Wei Dai, Jin Wen, Qionghui Yang, Ruixiang Chen

**Affiliations:** 1 Department of Pharmacy, The Third People’s Hospital of Yunnan Province, Kunming, 650011 Yunnan China; 2 Department of Ophthalmology, Southern theater general hospital, Guangzhou, 510010 China; 3 Department of Orthopaedics, The Third Affiliated Hospital, Sun Yat-sen University, Guangzhou, 510630 China; 4 College of Pharmacy, Dali University, Dali, 671003 Yunnan China

**Keywords:** Anxiety, Lorazepam, Pharmacovigilance, FAERS, Adverse events, Risk factors, Health policy

## Abstract

Lorazepam is extensively used to treat anxiety disorders and anxiety associated with depression. This study evaluates the safety of lorazepam based on real-world data from the U.S. Food and Drug Administration Adverse Event Reporting System (FAERS). Data were collected from January 2004 to June 2024. After standardizing the data, we quantified signals using four algorithms, including the Reporting Odds Ratio (ROR), the Proportional Reporting Ratio (PRR), the Bayesian Confidence Propagation Neural Network (BCPNN), and the Multi-Item Gamma Poisson Shrinker (MGPS) to quantize the signal by Bayesian analysis and disproportionation analysis. AE signals were predominantly involved psychiatric disorders, nervous system disorders, injury, poisoning and procedural complications, and cardiac disorders. Notably, new potential AE signals of clinical value were identified in this study, including tachycardia, rhabdomyolysis, neologism, phagophobia, pancreatic fibrosis, and pneumonia. Sex-stratified analysis showed that the risk of poisoning was more pronounced in females and the AEs of sedation were more pronounced in males. Age-stratified analysis demonstrated variations in AEs across different age groups.The findings of this study were consistent with clinical trials, and identified several new potential AE signals. In addition, there are gender and age differences in some AEs. These findings provide valuable insights into lorazepam in clinical practice.

**Supplementary Information:**

The online version contains supplementary material available at 10.1038/s41598-025-05680-z.

## Introduction

Lorazepam, a widely prescribed Benzodiazepines (BZDs), exhibits anticonvulsant, anxiolytic, and sedative effects. It was first introduced to the market in the United States in 1977. It is approved by the U.S. Food and Drug Administration (FDA) for the short-term relief of anxiety symptoms associated with anxiety disorders, anxiety-related insomnia, preanesthetic administration in adults to relieve anxiety or induce sedation or amnesia, and for the treatment of status epilepticus^1^. Lorazepam is particularly favored in hospitalized patients due to its rapid onset of action (1–3 min intravenously). Given its significant clinical efficacy, rapid onset, and lower cost compared to alternative drug classes, lorazepam is commonly used for acute-onset psychiatric conditions such as agitation, psychosis, and anxiety^2^. Currently, there is evidence that lorazepam is highly effective in treating catatonia, more or less immediately, and often saves the lives of many patients suffering from catatonia^3^.

BZDs accelerate the immobilization of γ-Aminobutyric Aci (GABA) on GABA_A_-type receptors, thereby increasing the inhibitory effects of GABA and reducing neuronal excitability enhance affinity^4^. They act as nonspecific inhibitors of central nervous system activity through orthomorphic modulation of the GABA_A_ receptor in the GABA system, functioning as central nervous system depressants^5^. Lorazepam is available in tablet form (0.5 mg, 1 mg, or 2 mg per tablet) and injectable form^6^. It also has some unique availability as an intramuscular (IM), immediate (STAT) or PRN dose parenterally^7^.

However, despite the significant clinical benefits of lorazepam, its widespread use inevitably leads to adverse effects in patients due to individual variability. Evidence suggests that alterations in the GABA neurotransmitter system increase susceptibility to adverse effects such as somnolence, confusion, ataxia, sedation, falls, impaired driving, and cognitive impairment^8^. Long-term use of BZDs not only increases the risk of dependence but also poses potential problems related to substance abuse and intoxication, which can result in serious consequences such as withdrawal delirium, seizures, and in severe cases, death^9^. In addition, there is evidence that long-term use of BZDs increases the risk of dementia^10,11^. Therefore, the risk of clinical use of lorazepam should not be ignored, and the safety of long-term use still needs to be further evaluated.

The FDA Adverse Event Reporting System (FAERS) database is a commonly used open data platform for exploring drug safety information, aiming to collect and analyze adverse events (AEs) occurring during the use of drugs and biologics, especially rare adverse reactions that can be captured. In this study, based on the FAERS database and employing data mining techniques, we conducted a retrospective pharmacovigilance analysis to detect the AEs signals of lorazepam using disproportionate analysis methods and analyze the potential associations between lorazepam and AEs in the real world, with the aim of providing more safety information about the clinical use of lorazepam and providing valuable insights for clinical practice.

## Materials and methods

### Data sources

The data used in this study were sourced from the FAERS database. This database contains reports of adverse reactions to various drugs and biologics submitted by healthcare providers, patients, and pharmaceutical manufacturers. The reports include the drug name, route of administration, descriptions and severities of AEs. The FAERS database of AE data is updated quarterly. For this study, data were collected from Q1(the first quarter) 2004 to Q2(the second quarter) 2024 (a total of 82 quarters), including patient demographics, administrative information, drug/biologic information, AEs, patient outcomes, source of report, start and end dates of drug therapy etc.

### Data processing

All data analyses were imported into SAS 9.4 and Microsoft Excel software for data cleaning and analysis. We utilized the Medical Dictionary for Regulatory Activities (MedDRA) Version 27.1 for the standardization and classification of AEs at both the Preferred Term (PT) and System Organ Class (SOC) levels. The keywords “lorazepam”, “ATIVAN”, “LORAZ”, “LOREEV XR”, “Témesta”, “Donix”, “Duralozam”, and “Laubeel” were used for screening, and the level of suspicion was limited to “primary suspect " drug. Limiting the primary suspected drug can make the research more targeted, avoid the interference of multiple drugs in the judgment of adverse reaction signals, and help to more accurately explore and analyze the potential association between specific drugs and adverse events. Thus, AE reports were retrieved where lorazepam was identified as the primary suspect drug.

**Fig. 1:**
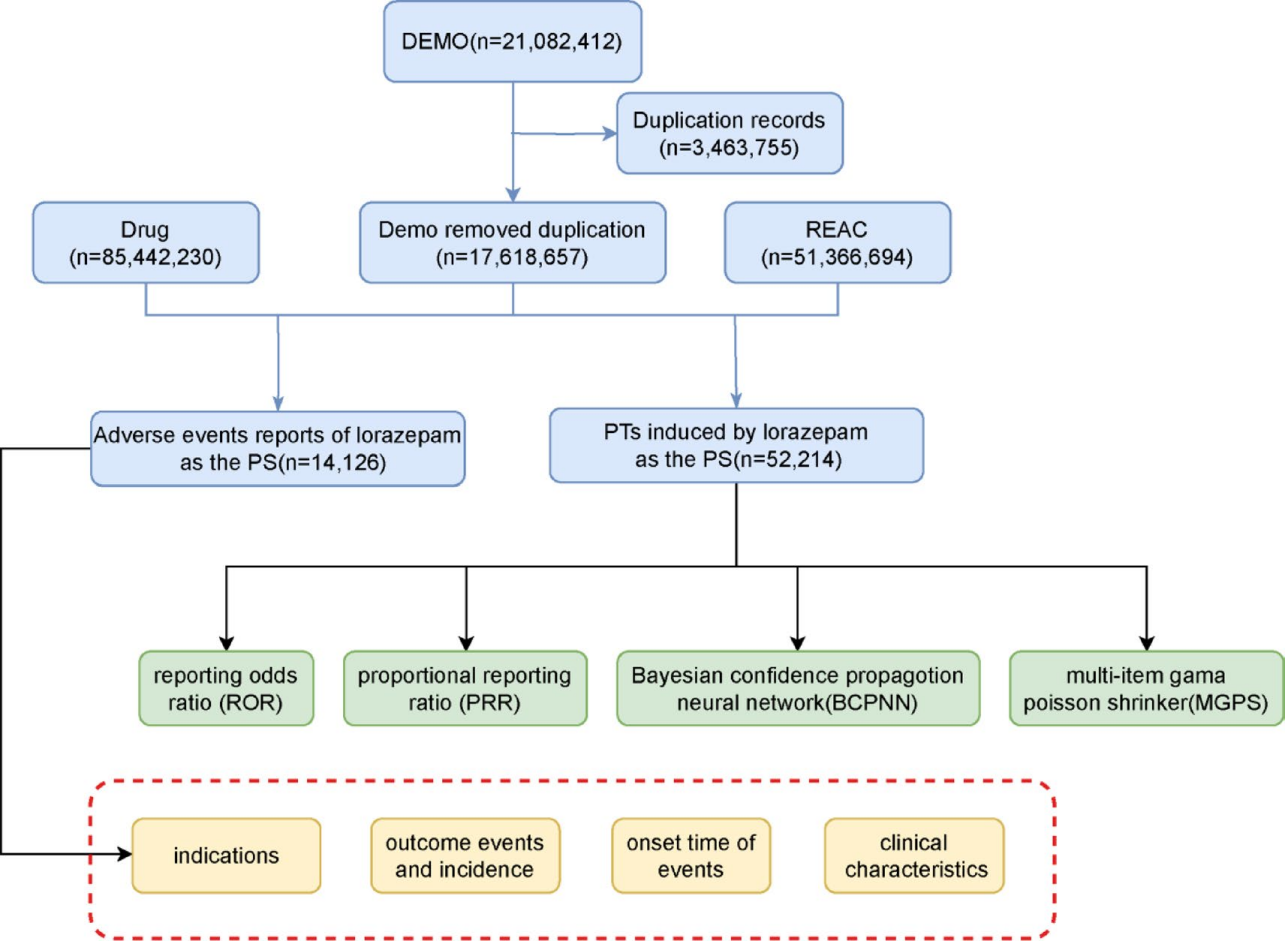
The flow diagram of selecting lorazepam-related AEs from the FAERS database.

Since there is a certain percentage of duplicate reports in the FAERS database, the reports were de-duplicated according to the FDA-recommended method for removing duplicate reports. The PRIMARYID, CASEID, and FDA_DT fields of the DEMO table are selected and sorted by CASEID, FDA_DT, and PRIMARYID. For reports with the same CASEID, the one with the largest FDA_DT value is retained, and for the same CASEID and FDA_DT the one with the largest PRIMARYID value is retained, ensuring only the most recent report remained. The comprehensive screening process is shown in Fig. 1.

### Data analysis

In this study, we mainly employed the proportional imbalance method^12^. That is, the proportion of a particular AE occurring in a specific drug in the database is significantly higher than the background frequency of the whole database, which is considered as proportional imbalance. To ensure sensitivity and credibility of our findings and minimize bias from relying on a single algorithm, we used four statistical methods to mine the signals of lorazepam-associated AEs in this study based on imbalance analysis and Bayesian analysis, including the Reporting Odds Ratio (ROR), the Proportional Reporting Ratio (PRR), the Bayesian Confidence Propagation Neural Network (BCPNN), and the Multi-Item Gamma Poisson Shrinker (MGPS). The ROR algorithm requires at least 3 cases (a ≥ 3) and a 95% confidence interval (CI) with a lower limit > 1, accounting for both event frequency and statistical significance^13^. The PRR algorithm requires a ≥ 3, PRR ≥ 2^14^, and a chi-squared test statistic (χ²) ≥ 4 to ensure proportional imbalance exceeds background rates. The BCPNN algorithm requires a ≥ 3, and the lower limit of 95% CI of the IC (IC025) > 0, leveraging Bayesian inference to handle sparse data. The MGPS algorithm requires a > 0 and the lower 5% confidence limit of the empirical Bayesian geometric mean (EBGM05) > 2, incorporating shrinkage estimation to stabilize rare event signals. A positive signal was defined when all four algorithms yielded positive results. All algorithms were based on the four-grid table of the proportional imbalance method (Table 1), and the formulas and judgment criteria of the four algorithms are detailed in Table 2. Data preprocessing, mining, statistical analysis and visualization were performed using R software version 4.4.3 and Microsoft Excel software.

**Table 1:** Proportional imbalance method four grid table.

	Drug-related ADEs	Non-drug-related ADEs	Total
Drug	a	b	a + b
Non-drug	c	d	c + d
Total	a + c	b + d	N = a + b + c + d

ADEs, adverse drug events; a is the number of cases with specific adverse events after drug is used; b is the number of cases with drug but no specific adverse events, c is the number of cases with specific adverse events without drug, and d is the number of cases without drug and no specific adverse events.

**Table 2:** ROR, PRR, BCPNN, and MGPS algorithms, equations, and criterias.

Algorithms	Equation	Criteria
ROR	$\:\text{R}\text{O}\text{R}=\frac{(\text{a}/\text{c})}{(\text{b}/\text{d})}=\frac{\text{a}\text{d}}{\text{b}\text{c}}$	a ≥ 3 95%CI (lower limit) > 1
	$$\:{95{\%}\text{C}\text{I}=\text{e}}^{\text{ln}\left(\text{R}\text{O}\text{R}\right)\pm\:1.96\sqrt{(\frac{1}{\text{a}}+\frac{1}{\text{b}}+\frac{1}{\text{c}}+\frac{1}{\text{d}})}}$$	
PRR	$\:\text{P}\text{R}\text{R}=\frac{\text{a}/(\text{a}+\text{b})}{\text{c}/(\text{c}+\text{d})}$	a ≥ 3 PRR ≥ 2 χ^2^ ≥ 4
	$$\:{{\upchi\:}}^{2}=\frac{(\text{a}+\text{b}+\text{c}+\text{d}){(\text{a}\text{d}-\text{b}\text{c})}^{2}}{(\text{a}+\text{b})(\text{c}+\text{d})(\text{a}+\text{c})(\text{b}+\text{d})}$$	
BCPNN	$\:\text{I}\text{C}={\text{l}\text{o}\text{g}}_{2}\frac{\text{a}(\text{a}+\text{b}+\text{c}+\text{d})}{(\text{a}+\text{b})(\text{a}+\text{c})}$	a ≥ 3 IC025 > 0
	$$\:{\upgamma\:}={{\upgamma\:}}_{11}\frac{(\text{a}+\text{b}+\text{c}+\text{d}+{\upalpha\:})(\text{a}+\text{b}+\text{c}+\text{d}+{\upbeta\:})}{(\text{a}+\text{b}+{{\upalpha\:}}_{1})(\text{a}+\text{c}+{{\upbeta\:}}_{1})}$$	
	$$\:\text{E}\left(\text{I}\text{C}\right)={\text{l}\text{o}\text{g}}_{2}\frac{(\text{a}+{{\upgamma\:}}_{11})(\text{a}+\text{b}+\text{c}+\text{d}+{\upalpha\:})(\text{a}+\text{b}+\text{c}+\text{d}+{\upbeta\:})}{(\text{a}+\text{b}+\text{c}+\text{d}+{\upgamma\:})(\text{a}+\text{b}+{{\upalpha\:}}_{1})(\text{a}+\text{c}+{{\upbeta\:}}_{1})}$$	
	$$\:\text{V}\left(\text{I}\text{C}\right)=\frac{1}{{\left(\text{l}\text{n}2\right)}^{2}}\left[\frac{\left(\text{a}+\text{b}+\text{c}+\text{d}\right)-\text{a}+{\upgamma\:}-{{\upgamma\:}}_{11}}{\left(\text{a}+{{\upgamma\:}}_{11}\right)\left(1+\text{a}+\text{b}+\text{c}+\text{d}+{\upgamma\:}\right)}+\frac{\left(\text{a}+\text{b}+\text{c}+\text{d}\right)-\left(\text{a}+\text{b}\right)+{\upalpha\:}-{{\upalpha\:}}_{1}}{\left(\text{a}+\text{b}+{{\upalpha\:}}_{1}\right)\left(1+\text{a}+\text{b}+\text{c}+\text{d}+{\upalpha\:}\right)}+\frac{\left(\text{a}+\text{b}+\text{c}+\text{d}\right)-\left(\text{a}+\text{c}\right)+{\upbeta\:}-{{\upbeta\:}}_{1}}{(\text{a}+\text{c}+{{\upbeta\:}}_{1})(1+\text{a}+\text{b}+\text{c}+\text{d}+{\upbeta\:})}\right]$$	
	$$\:\text{I}\text{C}025=\text{E}\left(\text{I}\text{C}\right)-2\sqrt{\text{V}\left(\text{I}\text{C}\right)}$$	
MGPS	$\:\text{E}\text{B}\text{G}\text{M}=\frac{\text{a}(\text{a}+\text{b}+\text{c}+\text{d})}{(\text{a}+\text{c})/(\text{a}+\text{b})}$	a > 0 EBGM05 > 2
	$$\:\text{E}\text{B}\text{G}\text{M}05={\text{e}}^{\text{ln}\left(\text{E}\text{B}\text{G}\text{M}\right)\pm\:1.96\sqrt{(\frac{1}{\text{a}}+\frac{1}{\text{b}}+\frac{1}{\text{c}}+\frac{1}{\text{d}})}}$$	

ROR, reporting odds ratio; a, number of cases with specific adverse events after drug is used; b, number of cases with drug but no specific adverse events, c, number of cases with specific adverse events without drug, d, number of cases without drug and no specific adverse events; CI, confidence interval; PRR, proportional reporting ratio; χ2chi-squared; BCPNN, Bayesian confidence propagation neural network; IC, information components; γ, γ_11_ are the Dicichlet distribution parameter; α_1_, α, β_1_, β are Beta distribution parameter; E(IC), the IC, expectations; V(IC), the variance of IC; IC025, the lower confidence interval of IC; MGPS, multi-item gamma Poisson shrinker; EBGM, empirical Bayes geometric mean; EBGM05, the lower limit of 95% CI of EBGM.

## Results

### Basic characteristics

**Fig. 2:**
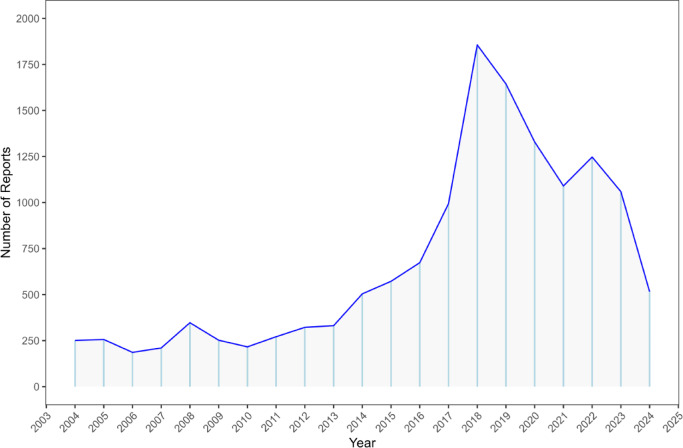
The number of ADEs reported yearly after the marketing of lorazepam. ADEs: Adverse drug events.

**Fig. 3:**
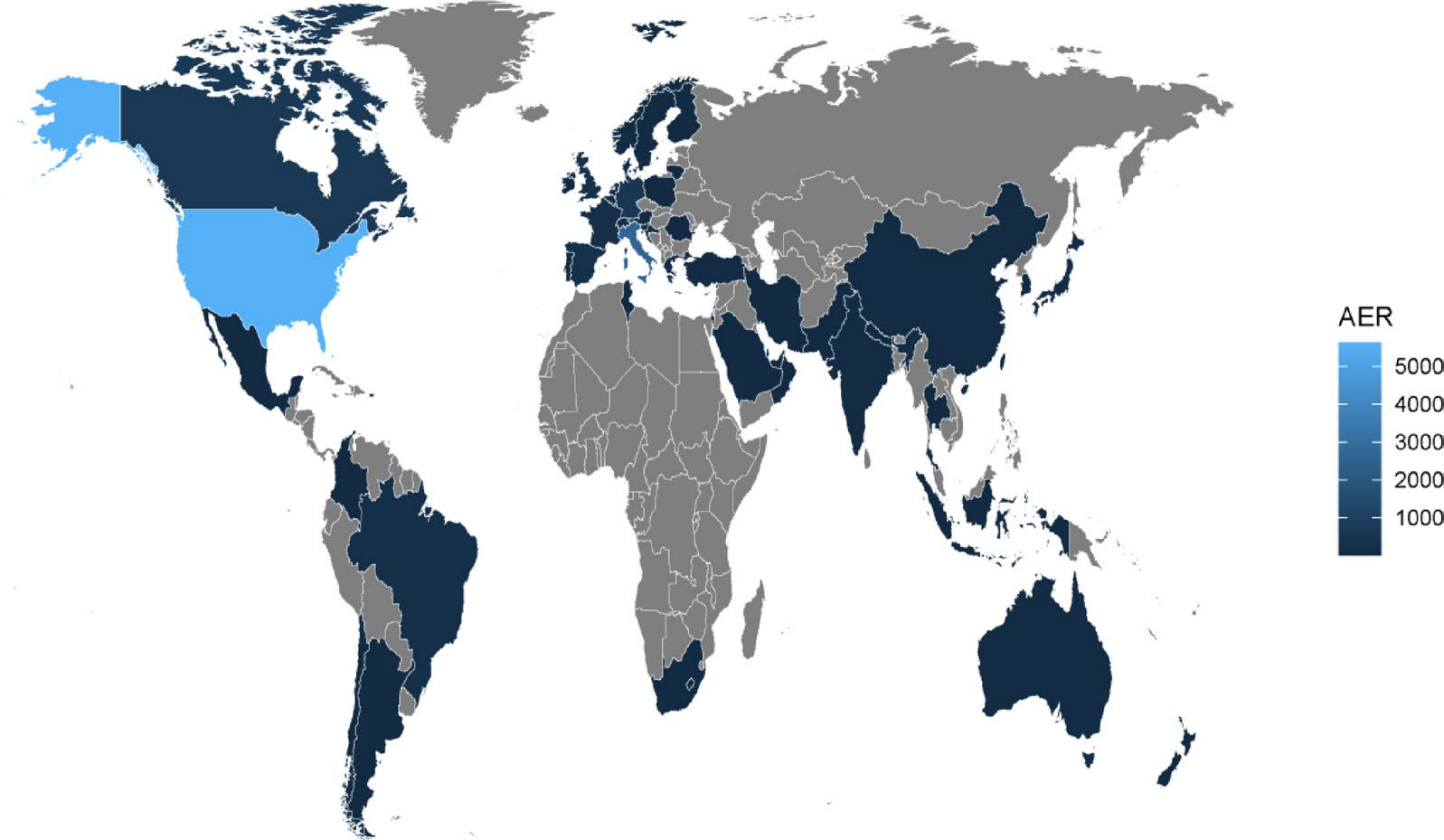
Geographic distribution of adverse event reports associated with lorazepam. AER: Adverse event report.

In this study, all the reported cases of lorazepam from Q1 2004 to Q2 2024 were extracted (*n* = 21,082,412), and a total of 14,126 reports were screened for lorazepam as the primary suspect. The number of reports from 2018 to 2023 exceeded 1,000 cases annually, with the highest number of reports in 2018 (13.14%). The quarterly distribution of reports is shown in Fig. 2. The proportion of reports lacking gender information was 7.86%, there were significantly more females (57.86%) than males (34.28%) reported. In terms of age, 25.51% of the data were missing, among the available data, 47.24% were 18–65 years old, 22.77% were over 65 years old, and 4.48% were under 18 years old. The most frequently reported country was the United States (47.16%), followed by Italy (22.20%), Canada (5.42%), Germany (4.92%), and the United Kingdom (2.88%), as illustrated in Fig. 3. Colors indicate the number of reports per country, gray represents countries with almost no or no reported cases. The primary reporters were physicians (29.29%), patients (27.74%), and pharmacists (20.21%). Among the clinical outcomes, except for unspecified serious AEs, hospitalization (37.03%) was the most common serious AE, followed by death (12.68%), life-threatening (6.47%), and disability (2.21%), as detailed in Table 3.

**Table 3:** Characteristics of reports with lorazepam from the FAERS database.

Characteristics	number	proportion
Gender		
Female	8173	57.86
Male	4843	34.28
Unknown	1110	7.86
Age		
< 18	633	4.48
18–65	6673	47.24
≥ 65	3216	22.77
Unknow	3604	25.51
Reported countries(Top five)		
United States	5616	47.16
Italy	2644	22.20
Canada	645	5.42
Germany	586	4.92
United Kingdom	343	2.88
Outcomes		
Other serious	5884	40.08
Hospitalization	5437	37.03
Death	1862	12.68
Life threatening	950	6.47
Disability	325	2.21
Required intervention to prevent permanent impairment/Damage	166	1.13
Congenital anomaly	58	0.40

### Signal detection

#### Based on SOC level

We conducted signal detection for lorazepam-associated AE at the SOC level, with further details provided in Table 4. A total of 25 SOC categories of AEs were identified. The data emphasized that the SOC meeting all four criteria simultaneously and was statistically significantly associated with lorazepam AE was the psychiatric disorders (*n* = 13177, ROR 5.4, PRR 4.29, IC 2.1, EBGM 4.27). This finding underscores that psychotic symptoms are considered to be a common AE of lorazepam, reflecting lorazepam’s use as an anti-anxiety drug. Significant SOC that met at least one of the four criteria were nervous system disorders (*n* = 7910, ROR 1.86, PRR 1.73,IC 0.79, EBGM 1.73); injury, poisoning and procedural complications (*n* = 5833, ROR 1.2, PRR 1.18, IC 0.24, EBGM 0.85), cardiac disorders (*n* = 1716, ROR 1.2, PRR 1.19, IC 0.25, EBGM 1.19), and ear and labyrinth disorders (*n* = 295, ROR 1.27, PRR 1.27, IC 0.35, EBGM 1.27). The remaining most frequently reported SOCs were general disorders and administration site conditions (*n* = 7905), gastrointestinal disorders (*n* = 2638), investigations (*n* = 2499), respiratory, thoracic and mediastinal disorders (*n* = 2322), and musculoskeletal and connective tissue disorders (*n* = 1258). In addition, metabolism and nutrition disorders (*n* = 795), infections and infestations (*n* = 784), eye disorders (*n* = 731), and immune system disorders (*n* = 544) were also common and noteworthy SOC categories.

**Table 4:** The signal strength of AEs of lorazepam at the SOC level in the FAERS database.

System organ class (SOC)	Case reports (*n* = 52214)	ROR (95%CI)	PRR ( 95%CI )	IC (IC025)	EBGM (EBGM05)
Psychiatric disorders	13,177	5.4(5.29, 5.51)*	4.29(4.21, 4.37)*	2.1(2.07)*	4.27(4.2)*
Nervous system disorders	7910	1.86(1.82, 1.91)*	1.73(1.7, 1.76)	0.79(0.76)*	1.73(1.7)
General disorders and administration site conditions	7905	0.82(0.8, 0.84)	0.85(0.83, 0.87)	-0.24(-0.28)	0.85(0.83)
Injury, poisoning and procedural complications	5833	1.2(1.17, 1.23)*	1.18(1.16, 1.2)	0.24(0.2)*	1.18(1.15)
Gastrointestinal disorders	2638	0.55(0.53, 0.57)	0.57(0.55, 0.59)	-0.8(-0.86)	0.57(0.56)
Investigations	2499	0.74(0.71, 0.77)	0.75(0.72, 0.78)	-0.42(-0.48)	0.75(0.72)
Respiratory, thoracic and mediastinal disorders	2322	0.9(0.86, 0.94)	0.91(0.88, 0.95)	-0.14(-0.2)	0.91(0.87)
Cardiac disorders	1716	1.2(1.14, 1.26)*	1.19(1.14, 1.24)	0.25(0.18)*	1.19(1.14)
Musculoskeletal and connective tissue disorders	1258	0.43(0.4, 0.45)	0.44(0.41, 0.47)	-1.18(-1.26)	0.44(0.42)
Skin and subcutaneous tissue disorders	988	0.33(0.31, 0.35)	0.34(0.32, 0.36)	-1.54(-1.64)	0.34(0.33)
Vascular disorders	825	0.7(0.66, 0.75)	0.71(0.67, 0.75)	-0.5(-0.6)	0.71(0.67)
Metabolism and nutrition disorders	795	0.68(0.64, 0.73)	0.69(0.64, 0.75)	-0.54(-0.64)	0.69(0.65)
Infections and infestations	784	0.27(0.25, 0.29)	0.28(0.26, 0.3)	-1.85(-1.95)	0.28(0.26)
Eye disorders	731	0.68(0.63, 0.73)	0.68(0.63, 0.74)	-0.55(-0.66)	0.68(0.64)
Immune system disorders	544	0.91(0.84, 0.99)	0.91(0.84, 0.98)	-0.13(-0.25)	0.91(0.85)
Renal and urinary disorders	518	0.52(0.47, 0.56)	0.52(0.48, 0.56)	-0.94(-1.06)	0.52(0.49)
Ear and labyrinth disorders	295	1.27(1.13, 1.43)*	1.27(1.13, 1.43)	0.35(0.18)*	1.27(1.15)
Hepatobiliary disorders	281	0.57(0.51, 0.64)	0.57(0.51, 0.64)	-0.81(-0.98)	0.57(0.52)
Blood and lymphatic system disorders	270	0.29(0.26, 0.33)	0.3(0.27, 0.34)	-1.76(-1.93)	0.3(0.27)
Surgical and medical procedures	218	0.3(0.26, 0.34)	0.3(0.26, 0.34)	-1.73(-1.92)	0.3(0.27)
Neoplasms benign, malignant and unspecified (incl cysts and polyps)	177	0.12(0.1, 0.14)	0.12(0.1, 0.14)	-3.02(-3.23)	0.12(0.11)
Pregnancy, puerperium and perinatal conditions	157	0.67(0.58, 0.79)	0.67(0.57, 0.78)	-0.57(-0.79)	0.67(0.59)
Reproductive system and breast disorders	137	0.31(0.26, 0.36)	0.31(0.26, 0.37)	-1.7(-1.94)	0.31(0.27)
Endocrine disorders	118	0.87(0.72, 1.04)	0.87(0.73, 1.04)	-0.21(-0.46)	0.87(0.75)
Congenital, familial and genetic disorders	118	0.71(0.59, 0.85)	0.71(0.6, 0.85)	-0.5(-0.76)	0.71(0.61)

*Indicates statistically significant signals in the algorithm; ROR, reporting odds ratio; CI, confidence interval; PRR, proportional reporting ratio; IC, information component; EBGM, empirical Bayesian geometric mean; IC025, the lower limit of 95% CI of the IC; EBGM05, the lower limit of 95% CI of EBGM.

#### Based on PT level

At the PT level, this study used four algorithms to analyze AEs and assess whether they met the various screening criteria, yielding 350 PTs. These PTs were ranked based on signal frequency, and the top 50 PTs with the highest associations are presented in Table 5. Besides drug abuse (*n* = 1772), our findings identified sopor (*n* = 1218), somnolence (*n* = 826), completed suicide (*n* = 768), suicide attempt (*n* = 653), toxicity to various agents (*n* = 630), and intentional overdose (*n* = 544) as the most common AEs, which generally align with the product labels and clinical trials.

**Table 5:** The top 50 signal strength of AEs of lorazepam ranked by the frequency at the PTs level in FAERS database.

SOC	PTs	Case reports (*n*=)	ROR (95%CI)	PRR ( 95%CI )	IC (IC025)	EBGM (EBGM05)
Psychiatric disorders	Drug abuse	1772	24.9(23.74, 26.12)	24.09(23.16, 25.05)	4.56(4.49)	23.54(22.61)
Psychiatric disorders	Sopor	1218	107.85(101.59, 114.48)	105.35(99.33, 111.73)	6.57(6.49)	95.25(90.61)
Nervous system disorders	Somnolence	826	4.67(4.36, 5)	4.61(4.35, 4.89)	2.2(2.1)	4.6(4.34)
Psychiatric disorders	Completed suicide	768	10.09(9.39, 10.84)	9.95(9.2, 10.76)	3.3(3.2)	9.86(9.29)
Psychiatric disorders	Suicide attempt	653	12.23(11.32, 13.22)	12.09(11.18, 13.08)	3.58(3.47)	11.96(11.2)
Injury, poisoning and procedural complications	Toxicity to various agents	630	4.42(4.08, 4.78)	4.38(4.05, 4.74)	2.13(2.01)	4.36(4.09)
Injury, poisoning and procedural complications	Intentional overdose	544	10.18(9.35, 11.08)	10.08(9.32, 10.9)	3.32(3.2)	9.99(9.31)
Psychiatric disorders	Confusional state	541	3.74(3.44, 4.08)	3.72(3.44, 4.02)	1.89(1.77)	3.71(3.45)
Psychiatric disorders	Drug dependence	480	3.06(2.79, 3.34)	3.04(2.76, 3.35)	1.6(1.47)	3.03(2.81)
Psychiatric disorders	Agitation	477	7.15(6.53, 7.83)	7.09(6.43, 7.82)	2.82(2.69)	7.05(6.53)
Psychiatric disorders	Intentional self-injury	457	25.92(23.61, 28.46)	25.71(23.31, 28.36)	4.65(4.51)	25.08(23.19)
General disorders and administration site conditions	Withdrawal syndrome	333	8.75(7.85, 9.75)	8.7(7.89, 9.6)	3.11(2.95)	8.63(7.88)
Cardiac disorders	Tachycardia	332	4.22(3.79, 4.71)	4.2(3.81, 4.63)	2.07(1.91)	4.19(3.83)
Psychiatric disorders	Delirium	305	10.32(9.22, 11.56)	10.27(9.13, 11.55)	3.35(3.18)	10.17(9.25)
General disorders and administration site conditions	Drug withdrawal syndrome	298	3.45(3.08, 3.87)	3.44(3.06, 3.87)	1.78(1.61)	3.43(3.12)
Nervous system disorders	Coma	293	6.9(6.15, 7.74)	6.87(6.11, 7.73)	2.77(2.61)	6.83(6.2)
Psychiatric disorders	Suicidal ideation	279	3.4(3.02, 3.83)	3.39(3.01, 3.81)	1.76(1.59)	3.38(3.06)
Nervous system disorders	Sedation	274	12.8(11.36, 14.42)	12.73(11.32, 14.32)	3.65(3.48)	12.58(11.39)
Nervous system disorders	Depressed level of consciousness	267	7.53(6.67, 8.49)	7.49(6.66, 8.42)	2.9(2.72)	7.45(6.73)
Cardiac disorders	Cardiac arrest	247	3.28(2.9, 3.72)	3.27(2.91, 3.68)	1.71(1.53)	3.27(2.94)
Injury, poisoning and procedural complications	Poisoning	239	20.9(18.38, 23.76)	20.81(18.14, 23.87)	4.35(4.17)	20.4(18.32)
Psychiatric disorders	Bradyphrenia	232	39.51(34.64, 45.06)	39.34(34.3, 45.13)	5.24(5.05)	37.86(33.92)
Respiratory, thoracic and mediastinal disorders	Respiratory arrest	215	8.2(7.17, 9.38)	8.17(7.12, 9.37)	3.02(2.83)	8.11(7.25)
Psychiatric disorders	Hallucination	212	3.26(2.85, 3.73)	3.25(2.83, 3.73)	1.7(1.5)	3.24(2.89)
Nervous system disorders	Speech disorder	204	4.31(3.75, 4.94)	4.3(3.75, 4.93)	2.1(1.9)	4.28(3.81)
General disorders and administration site conditions	Drug ineffective for unapproved indication	198	4.33(3.76, 4.98)	4.32(3.77, 4.96)	2.1(1.9)	4.3(3.83)
Psychiatric disorders	Aggression	195	4.25(3.69, 4.9)	4.24(3.7, 4.86)	2.08(1.88)	4.23(3.76)
Psychiatric disorders	Disorientation	194	5.32(4.62, 6.13)	5.31(4.63, 6.09)	2.4(2.2)	5.28(4.69)
Nervous system disorders	Dysarthria	172	5.07(4.36, 5.89)	5.06(4.33, 5.92)	2.33(2.12)	5.04(4.44)
Injury, poisoning and procedural complications	Medication error	151	3.08(2.62, 3.61)	3.07(2.62, 3.59)	1.62(1.39)	3.06(2.68)
Psychiatric disorders	Panic attack	148	4.54(3.87, 5.34)	4.53(3.87, 5.3)	2.18(1.94)	4.52(3.95)
Psychiatric disorders	Nervousness	148	3.02(2.57, 3.55)	3.01(2.57, 3.52)	1.59(1.36)	3.01(2.63)
Respiratory, thoracic and mediastinal disorders	Respiratory depression	148	13.44(11.42, 15.81)	13.4(11.46, 15.67)	3.73(3.49)	13.24(11.55)
Psychiatric disorders	Restlessness	137	4.14(3.5, 4.89)	4.13(3.46, 4.93)	2.04(1.8)	4.12(3.58)
Nervous system disorders	Unresponsive to stimuli	137	6.05(5.12, 7.16)	6.04(5.06, 7.21)	2.59(2.35)	6.01(5.22)
Psychiatric disorders	Psychotic disorder	131	4.94(4.16, 5.86)	4.93(4.13, 5.88)	2.3(2.05)	4.91(4.25)
Nervous system disorders	Cognitive disorder	131	3.19(2.68, 3.78)	3.18(2.67, 3.79)	1.67(1.42)	3.17(2.75)
Nervous system disorders	Neuroleptic malignant syndrome	123	11.74(9.82, 14.03)	11.71(9.82, 13.97)	3.53(3.28)	11.59(9.98)
Psychiatric disorders	Catatonia	122	27.84(23.25, 33.34)	27.78(23.29, 33.14)	4.76(4.5)	27.04(23.26)
Cardiac disorders	Cardio-respiratory arrest	119	3.04(2.54, 3.64)	3.03(2.54, 3.61)	1.6(1.34)	3.03(2.6)
Musculoskeletal and connective tissue disorders	Rhabdomyolysis	111	3.01(2.49, 3.62)	3(2.51, 3.58)	1.58(1.31)	3(2.56)
Nervous system disorders	Hypokinesia	107	8.12(6.71, 9.82)	8.1(6.66, 9.85)	3.01(2.73)	8.04(6.86)
Injury, poisoning and procedural complications	Accidental overdose	107	3.51(2.9, 4.25)	3.51(2.89, 4.27)	1.81(1.53)	3.5(2.98)
Psychiatric disorders	Mental status changes	105	4.19(3.46, 5.08)	4.18(3.44, 5.09)	2.06(1.78)	4.17(3.55)
Nervous system disorders	Psychomotor hyperactivity	103	6.7(5.52, 8.13)	6.69(5.5, 8.14)	2.73(2.45)	6.65(5.65)
Psychiatric disorders	Fear	100	3.91(3.21, 4.76)	3.9(3.21, 4.74)	1.96(1.68)	3.89(3.3)
Nervous system disorders	Bradykinesia	99	21.34(17.48, 26.04)	21.3(17.51, 25.91)	4.38(4.1)	20.87(17.66)
Psychiatric disorders	Nightmare	97	3.08(2.52, 3.76)	3.08(2.53, 3.75)	1.62(1.33)	3.07(2.6)
Infections and infestations	Pneumonia aspiration	95	4.32(3.53, 5.28)	4.31(3.54, 5.24)	2.1(1.81)	4.3(3.63)
Eye disorders	Mydriasis	94	8.34(6.8, 10.21)	8.32(6.84, 10.12)	3.05(2.75)	8.26(6.97)

ROR, reporting odds ratio; CI, confidence interval; PRR, proportional reporting ratio; IC, information component; EBGM, empirical Bayesian geometric mean; IC025, the lower limit of 95% CI of the IC; EBGM05, the lower limit of 95% CI of EBGM; PT: preferred term.

Statistically, the PTs meeting all four screening criteria were ranked using the most sensitive ROR algorithm, as detailed in Table 6. AE with significant potential signals included kluver-bucy syndrome (ROR = 491.43, PRR = 491.39, IC = 8.36, EBGM = 327.92), urticaria physical (ROR = 446.76, PRR = 446.71, IC = 8.26, EBGM = 307.43), maximal voluntary ventilation abnormal (ROR = 421.21, PRR = 421.19, IC = 8.21, EBGM = 295.13), neologism (ROR = 218.41, PRR = 218.39, IC = 7.48, EBGM = 178.87), withdrawal catatonia (ROR = 211.91, PRR = 211.8, IC = 7.45, EBGM = 174.43), and others. In addition, it is worth noting that compared with the latest package insert released by the FDA, this study identified new potential AE signals of clinical value, such as tachycardia, rhabdomyolysis, kluver-bucy syndrome, maximal voluntary ventilation abnormal, neologism, phagophobia, pancreatic fibrosis, induced abortion failed, floppy infant, congenital pneumonia, and mitochondrial encephalomyopathy, among others.

**Table 6:** The top 50 signal strength of AEs of lorazepam ranked by the ROR at the PTs level in FAERS database.

SOC	PTs	Case reports (*n*=)	ROR (95%CI)	PRR (95%CI)	IC (IC025)	EBGM (EBGM05)
Nervous system disorders	Kluver-Bucy syndrome	5	491.43(167.96, 1437.84)	491.39(167.21, 1444.1)	8.36(7)	327.92(133.55)
Skin and subcutaneous tissue disorders	Urticaria physical	5	446.76(155.22, 1285.88)	446.71(155.01, 1287.31)	8.26(6.92)	307.43(126.93)
Investigations	Maximal voluntary ventilation abnormal	3	421.21(108.92, 1628.95)	421.19(108.93, 1628.61)	8.21(6.55)	295.13(95.17)
Psychiatric disorders	Neologism	4	218.41(73.91, 645.38)	218.39(74.31, 641.8)	7.48(6.09)	178.87(72.25)
Psychiatric disorders	Withdrawal catatonia	25	211.91(137.53, 326.49)	211.8(137.61, 325.98)	7.45(6.84)	174.43(121.49)
Investigations	Therapeutic agent urine negative	4	206.92(70.39, 608.25)	206.9(70.4, 608.04)	7.42(6.03)	171.09(69.41)
Psychiatric disorders	Phagophobia	4	126.82(44.76, 359.28)	126.81(44.88, 358.34)	6.81(5.46)	112.43(47.04)
Investigations	Protein C increased	8	124.82(59.81, 260.48)	124.8(59.26, 262.83)	6.79(5.79)	110.85(59.89)
Investigations	Osmolar gap increased	9	117.95(59.08, 235.51)	117.93(59.39, 234.18)	6.72(5.77)	105.4(59.1)
Psychiatric disorders	Sopor	1218	107.85(101.59, 114.48)	105.35(99.33, 111.73)	6.57(6.49)	95.25(90.61)
Investigations	Electrocardiogram j wave	4	103.46(36.92, 289.89)	103.45(36.61, 292.33)	6.55(5.21)	93.69(39.56)
Investigations	Osmolar gap abnormal	4	95.89(34.34, 267.71)	95.88(34.6, 265.68)	6.45(5.12)	87.45(37.04)
Gastrointestinal disorders	Pancreatic fibrosis	3	79.69(24.57, 258.46)	79.68(24.58, 258.27)	6.21(4.72)	73.78(27.57)
Investigations	Coma scale	4	75.6(27.34, 209.04)	75.6(27.28, 209.49)	6.13(4.82)	70.27(30)
Nervous system disorders	Muscle tension dysphonia	4	71.48(25.9, 197.25)	71.47(25.79, 198.04)	6.06(4.74)	66.7(28.53)
Skin and subcutaneous tissue disorders	Solar urticaria	7	67.45(31.36, 145.08)	67.45(31.41, 144.86)	5.98(4.95)	63.18(33.29)
Injury, poisoning and procedural complications	Induced abortion failed	8	66.08(32.3, 135.2)	66.07(31.99, 136.44)	5.95(4.98)	61.97(34.04)
Musculoskeletal and connective tissue disorders	Floppy infant	11	65.93(35.81, 121.41)	65.92(35.9, 121.03)	5.95(5.11)	61.84(37.1)
Psychiatric disorders	Thought insertion	4	65.52(23.81, 180.29)	65.52(23.65, 181.55)	5.94(4.63)	61.49(26.36)
Surgical and medical procedures	Patient restraint	4	60.48(22.03, 166.02)	60.48(21.83, 167.59)	5.83(4.52)	57.03(24.5)
Injury, poisoning and procedural complications	Complicated fracture	3	57.81(18.04, 185.24)	57.81(18.19, 183.75)	5.77(4.31)	54.65(20.63)
Investigations	Osmolar gap	3	54.6(17.07, 174.64)	54.6(17.18, 173.54)	5.69(4.23)	51.78(19.57)
Investigations	Benzodiazepine drug level increased	4	49.76(18.22, 135.89)	49.76(18.31, 135.21)	5.57(4.27)	47.41(20.46)
Skin and subcutaneous tissue disorders	Urticaria thermal	6	47.95(21.13, 108.81)	47.94(21.05, 109.2)	5.52(4.42)	45.76(23.05)
Congenital, familial and genetic disorders	Congenital pneumonia	4	47.37(17.37, 129.19)	47.36(17.43, 128.69)	5.5(4.2)	45.23(19.54)
Psychiatric disorders	Confabulation	11	45.62(24.92, 83.51)	45.61(24.84, 83.74)	5.45(4.61)	43.64(26.31)
Congenital, familial and genetic disorders	Mitochondrial encephalomyopathy	3	45.36(14.26, 144.33)	45.36(14.27, 144.18)	5.44(3.99)	43.4(16.48)
Psychiatric disorders	Somatic hallucination	3	40.39(12.73, 128.16)	40.39(12.71, 128.38)	5.28(3.83)	38.83(14.78)
Psychiatric disorders	Bradyphrenia	232	39.51(34.64, 45.06)	39.34(34.3, 45.13)	5.24(5.05)	37.86(33.92)
Nervous system disorders	Drop attacks	20	37.17(23.78, 58.09)	37.16(23.68, 58.33)	5.16(4.53)	35.84(24.66)
Psychiatric disorders	Cotard’s syndrome	3	36.4(11.5, 115.24)	36.4(11.45, 115.7)	5.13(3.69)	35.13(13.4)
Psychiatric disorders	Malignant catatonia	7	35.83(16.86, 76.19)	35.83(17.01, 75.46)	5.11(4.09)	34.61(18.41)
Psychiatric disorders	Echolalia	9	34.29(17.64, 66.65)	34.28(17.6, 66.75)	5.05(4.14)	33.16(19.01)
Respiratory, thoracic and mediastinal disorders	Pneumonitis aspiration	6	32.95(14.6, 74.32)	32.94(14.46, 75.03)	5(3.91)	31.91(16.15)
Psychiatric disorders	Delirium tremens	12	31.71(17.84, 56.35)	31.7(17.96, 55.96)	4.94(4.14)	30.74(19)
Respiratory, thoracic and mediastinal disorders	Nasal flaring	3	29.19(9.26, 92.04)	29.19(9.18, 92.78)	4.83(3.39)	28.38(10.86)
Injury, poisoning and procedural complications	Administration related reaction	4	29.12(10.77, 78.72)	29.12(10.72, 79.12)	4.82(3.54)	28.31(12.32)
Nervous system disorders	Decorticate posture	3	28.91(9.17, 91.12)	28.91(9.1, 91.89)	4.81(3.37)	28.11(10.75)
Psychiatric disorders	Agoraphobia	35	28.54(20.39, 39.94)	28.52(20.44, 39.8)	4.79(4.32)	27.75(20.94)
Investigations	Blood osmolarity increased	8	28.39(14.06, 57.33)	28.38(14.01, 57.47)	4.79(3.83)	27.61(15.34)
Respiratory, thoracic and mediastinal disorders	Respiratory fatigue	6	28.35(12.59, 63.84)	28.35(12.69, 63.32)	4.79(3.7)	27.58(13.99)
Psychiatric disorders	Catatonia	122	27.84(23.25, 33.34)	27.78(23.29, 33.14)	4.76(4.5)	27.04(23.26)
General disorders and administration site conditions	Alcohol interaction	48	27.29(20.48, 36.36)	27.27(20.32, 36.59)	4.73(4.32)	26.56(20.89)
Endocrine disorders	Myxoedema	5	27.15(11.17, 66.02)	27.15(11.24, 65.59)	4.72(3.55)	26.45(12.57)
Musculoskeletal and connective tissue disorders	Myoglobinaemia	3	27.05(8.59, 85.18)	27.05(8.51, 85.98)	4.72(3.28)	26.35(10.09)
Congenital, familial and genetic disorders	Newborn persistent pulmonary hypertension	6	26.81(11.91, 60.32)	26.8(12, 59.86)	4.71(3.62)	26.12(13.25)
Psychiatric disorders	Psychomotor retardation	64	26.51(20.68, 33.99)	26.48(20.52, 34.16)	4.69(4.33)	25.81(20.97)
Psychiatric disorders	Intentional self-injury	457	25.92(23.61, 28.46)	25.71(23.31, 28.36)	4.65(4.51)	25.08(23.19)
Nervous system disorders	Slow speech	52	25.83(19.61, 34.03)	25.81(19.62, 33.96)	4.65(4.26)	25.18(19.99)
Infections and infestations	Intrauterine infection	3	25.64(8.15, 80.67)	25.64(8.23, 79.91)	4.64(3.21)	25.01(9.58)

ROR, reporting odds ratio; CI, confidence interval; PRR, proportional reporting ratio; IC, information component; EBGM, empirical Bayesian geometric mean; IC025, the lower limit of 95% CI of the IC; EBGM05, the lower limit of 95% CI of EBGM; PT: preferred term.

#### Onset time of events

**Fig. 4:**
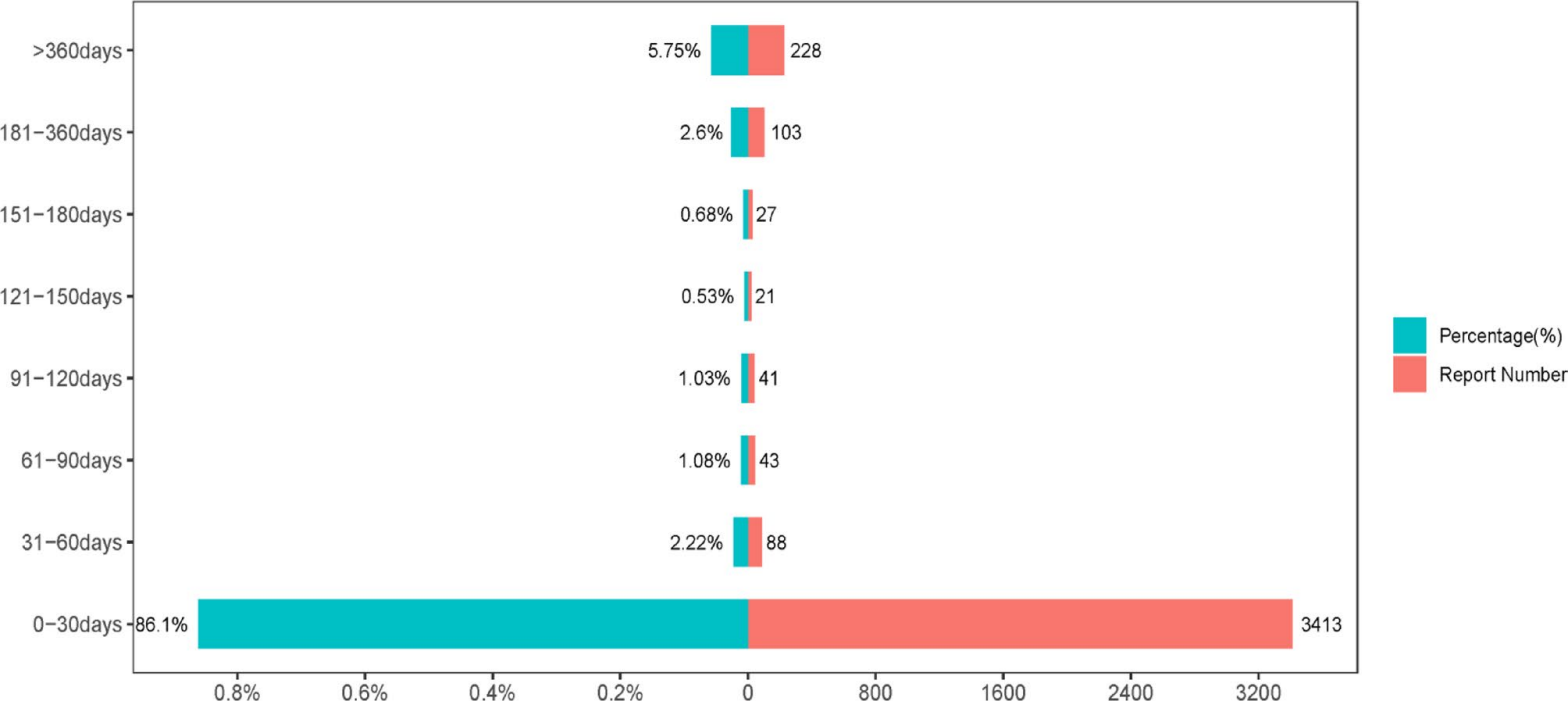
Time to onset of lorazepam related adverse events.

We collected the time of onset for lorazepam-related AEs, as shown in Fig. 4. This analysis excludes reports lacking time-of-onset information and includes a total of 3964 AEs. The data reveal that the vast majority of cases were reported within the first month after lorazepam administration, with a total of 3413 cases, accounting for 86.10%. Beyond this period, the onset time of events 2.22% of reports were recorded between 31 and 60 days, 0.53% between 121 and 150 days. Notably, a slight rebound was observed in the 181–360 days with 103 cases, accounting for 2.60% while another 5.75% occurred after 360 days. This makes us realize that vigilant monitoring of potential AEs in the early stages of drug administration and during long-term use is critical, and that timely recognition and intervention are necessary for optimal outcomes.

### Subgroup analysis

#### Gender-stratified risk signals

**Fig. 5:**
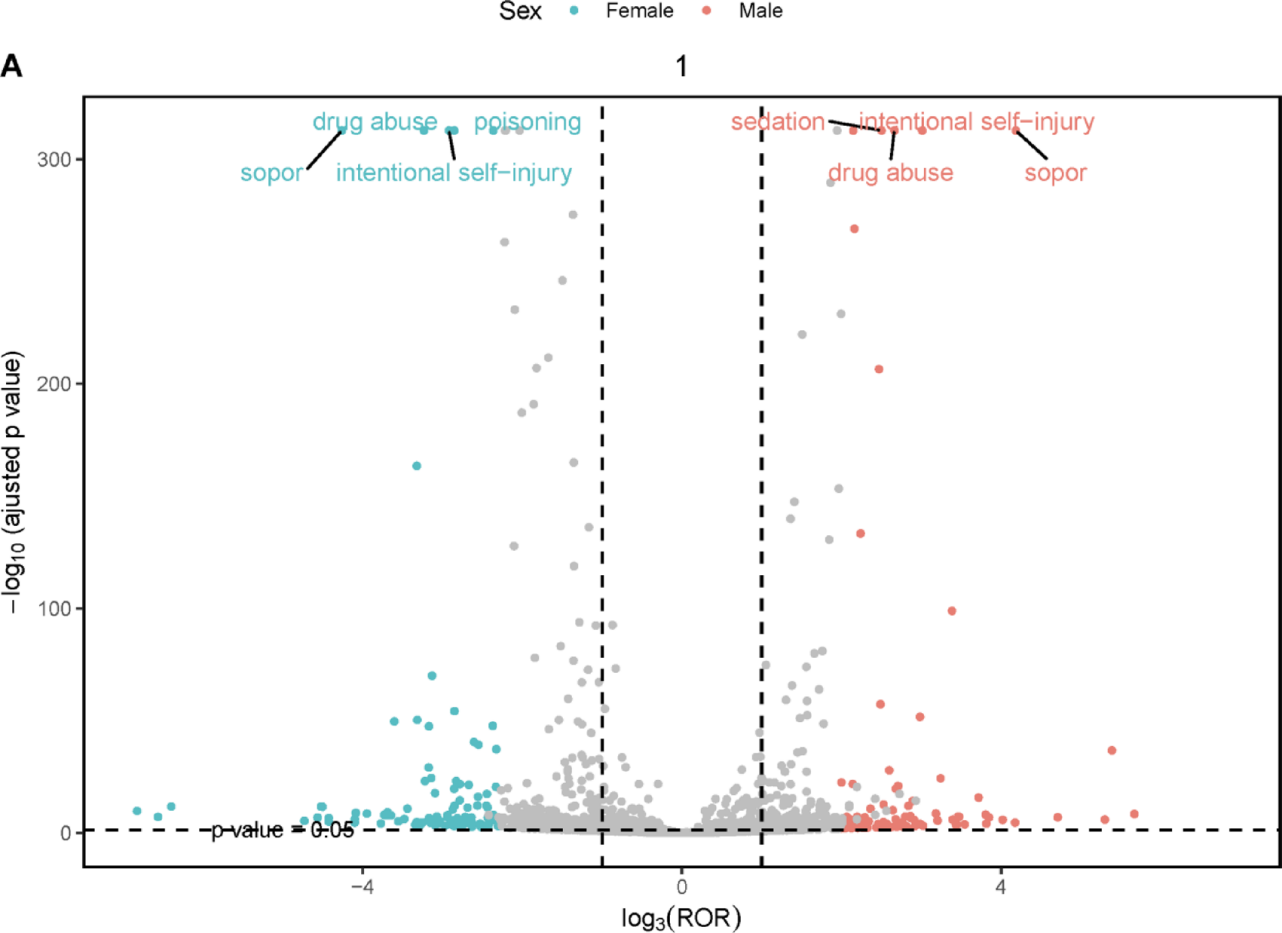
Gender-stratified risk volcano plot for lorazepam. ROR, reporting odds ratio.

We conducted an in-depth analysis of the gender-stratified AE signals for lorazepam using four statistical methods to evaluate PT. 7.86% of adverse event reports (1110 out of 14,126 cases) had missing gender information. The detailed results are shown in Supplementary Tables 1 and 2. “Volcano plots” were plotted for the visual presentation of the signal results, and our analyses revealed the level of gender-stratified AE for the lorazepam. The volcano plot was taken as a scale with -log10P values on the vertical axis and log3ROR values on the horizontal axis, as illustrated in Fig. 5. Each point on the plot represents an AE, with red points indicating potential AEs more likely to occur in male patients and green points representing those more likely in female patients. Sopor, drug abuse, and intentional self-injury were common in both females and males. Notably, poisoning was a more pronounced risk in the female population, while sedation was more prevalent in males. These findings underscore the importance of considering gender differences in clinical management.

#### Age-stratified risk signals

We employed four statistical methods to analyze PTs under 18 years of age, patients 18–65 years of age, and aged 65 years or older. The results are presented in Supplementary Tables S3–S5.We ranked the number of cases in descending order, the five most common PTs in patients younger than 18 years of age as drug abuse (*n* = 65), sopor (*n* = 49), somnolence (*n* = 43), agitation (*n* = 38), and suicide attempt (*n* = 33). For patients aged 18–65 years, the top five PTs were drug abuse (*n* = 1,297), sopor (*n* = 710), completed suicide (*n* = 556), toxicity to various agents (*n* = 428), and somnolence (*n* = 403). For patients over 65 years of age, the top five PTs were sopor (*n* = 347), drug abuse (*n* = 290), confusional state (*n* = 237), somnolence (*n* = 196), and completed suicide (*n* = 170). These findings indicate that there are distinct differences in AEs across different age groups, although drug abuse, sopor, and somnolence were common symptoms observed in all three age groups.

## Discussion

The overall therapeutic effect of a drug is determined by the balance between its efficacy and safety. Randomized controlled trials are the gold standard for determining the efficacy of drugs, but they are insufficient for evaluating adverse drug reactions (ADRs). It is of great significance to improve the rational use of drugs in clinics by fully exploring AEs through post-marketing safety monitoring, conducting continuous pharmacovigilance analysis, and providing decision support for individualized drug use guidance. The FAERS database has been publicly accessible since 2004 and has a large amount of information, which can effectively support post-marketing safety risk monitoring and analysis. In this study, a systematic investigation of all AEs related to lorazepam was conducted by in-depth analysis of the FAERS database from Q1 2004 to Q2 2024. Through an exhaustive and systematic review of worldwide reports on the harmful effects of the drug, the study not only confirmed known existing safety information but also identified new potential risks. As the prevalence of patients with anxiety, depression, and epilepsy increases, the clinical use of lorazepam is expected to expand accordingly. The findings from this comprehensive analysis provide more detailed and rationalized recommendations for healthcare practitioners and policy makers.

Patients often use multiple drugs simultaneously, which makes the attribution of adverse reactions complex. By limiting the primary suspected drug, the interference of other drugs can be excluded to a certain extent, and the statistical association between the target drug and adverse events can be presented more clearly. Generally, it is believed that the primary suspected drug has a higher possibility and greater impact on the occurrence of adverse events, while the contribution of secondary suspected drugs to adverse events is relatively small. Although it cannot completely determine the causal relationship, it can provide valuable clues for further research. While our analysis focused on cases where lorazepam was the primary suspect drug to ensure high specificity of signal detection, future studies may benefit from including secondary suspect or concomitant drug reports to explore broader drug-event associations.

In this study, we extracted 14,126 reports of lorazepam-related AEs from the FAERS database and analyzed their clinical characteristics. Despite a portion of the data lacking age-specific details, the results indicated that the incidence of AEs was significantly higher in female patients than in males. Although anxiety and depressive disorders are more prevalent in females than in males, there is a paucity of research on the potential impact of gender on the treatment of these disorders. Considering the relatively high prevalence of anxiety and depression among young women^15^is consistent with our findings. The age distribution of the reports was predominantly adults aged 18–65 years and older adults aged 65 years and above, consistent with the target population of the drug. Reports were mainly submitted by healthcare professionals, including physicians and pharmacists, but also from a certain number of consumers, suggesting that the data are reasonable and credible. However, the majority of reports came from the United States, with potential geographic bias, while there was limited data from Asia, which is a notable limitation. Recognizing the impact of regional differences can help target proposed interventions. Additionally, the FAERS database may be subject to data incompleteness and reporting bias, so addressing these issues will be crucial for improving post-marketing surveillance. We emphasize the need for ongoing monitoring and the need for further in-depth research.

In our investigation of lorazepam-related AEs, we analyzed signals from 25 systemic SOCs using four pharmacovigilance algorithms (ROR, PRR, BCPNN, and MGPS), as detailed in Table 4. The four algorithms consistently identified positive signals in the psychiatric disorders, reflecting lorazepam’s utility as an anxiolytic. These findings are consistent with data reported in the package insert and emphasize the need for vigilant monitoring of these particular AEs. Notably, the most common SOC was psychiatric disorders, with 13,177 cases (93.28% of all AE reports). The clinical signs were drug abuse, sopor, completed suicide, suicide attempt, confusional state, and drug dependence. These reactions emphasize the importance of careful and supervised drug administration. Nervous system disorders accounted for 56.00% of reports and general disorders and administration site conditions were also very common, accounting for 55.96% of the reports. Other notable SOCs included injury, poisoning and procedural complications (41.29%), gastrointestinal disorders (18.67%), investigations (17.79%) and respiratory, thoracic and mediastinal disorders (16.44%), reflecting the wide range of effects of the drug on multiple organ systems. This comprehensive profile requires multidisciplinary analysis. Overall, the consistent strong signals in both the psychiatric disorders and nervous system disorders call for intensified scrutiny and further research to better understand underlying mechanisms and optimize patient management strategies.

This study covers most of the AE signals and SOCs of lorazepam, consistent with the common side effects listed on the product label. GABA is a naturally occurring inhibitory neurotransmitter in the central nervous system (CNS) that reduces neuronal excitability^16^. GABA binds to neuronal GABA receptors, which makes the neuron less prone to generating action potentials or releasing the neurotransmitters. By targeting specific GABA_A_ receptor subtypes, selective states of the CNS such as alertness, anxiety, mood, pain perception, and memory can receive specific pharmacological modulation^17^. BZDs bind to GABA_A_ receptors, eliciting positive receptor modulation and enhanced GABA activity. Thus, sedative or anxiolytic effects are observed^18^. The anxiolytic effects at low doses and the sedative/hypnotic effects at high doses are the result of the dose-dependent action of BZDs on GABA receptors^19^. Improvements in insomnia when BZDs are used to treat anxiety and impaired sleep can be attributed, at least in part, to the direct sedative effects of the drugs. Some studies have demonstrated that cravings are prevalent during and after discontinuation in long-term BZDs users, but the severity of cravings decreases to negligible levels over time^20^. Anxiety and depression are the most prevalent psychiatric disorders^21^and because of the disorders themselves, the progression to severity can lead to suicidal thoughts. Effective treatment of these conditions can mitigate the risk of suicide. Physicians must conduct thorough psychological assessments and predictions to manage symptoms based on the patient’s condition, while family support is crucial for enhancing treatment adherence and confidence.

There are several reasons for the inherent pattern of physicians in prescribing BZDs. These factors stem from both internal limitations and external challenges. Internally, some physicians may lack awareness of the AEs and appropriate timing of BZD use, leading to an overestimation of the benefit-to-risk ratio. Additionally, there is often insufficient training in adjusting medication regimens to address emerging issues during treatment. Externally, patient resistance to medication adjustments, limited time and resources within the healthcare system, scarcity of psychological support, and inadequate regular medication reviews further exacerbate the situation.

The abuse of BZDs poses a significant challenge to the public health, with far-reaching consequences for individuals for individuals and society. The urgent need for comprehensive, evidence-based prevention, intervention and regulatory strategies is emphasized. Continuing efforts to conduct research and develop targeted approaches to effectively address drug abuse are important, and the present study may inform future initiatives aimed at curbing drug abuse.

However, BZDs may adversely affect neurocognitive function primarily in elderly patients^22^. GABAergic neurons are complex in regulating memory and learning characteristic variables. Long-term use of intermediate-acting BZDs, such as lorazepam, may produce amnestic effects due to attenuation of synaptic plasticity and impairment of recognition memory. However, BZDs may be protective against the development of Alzheimer’s disease (AD) by reducing tau phosphorylation, neuroinflammation, and neuropathological progression in AD. On the other hand, other studies indicate that long-term use of BZDs is not associated with the development of AD. In conclusion, there is a controversy about the use of BZDs and the risk of AD progression^23^.

In the literature, it has been reported that certain BZDs may cause alterations in electrocardiograph (ECG) parameters^24^. However, several studies have reported that lorazepam does not directly affect ECG parameters, including PR prolongation, QRS widening, and QTc interval^25^. BZDs are anxiolytics that have been found to be useful in treating patients with vertigo. These drugs act through GABA receptors, potentiate the effects of endogenous GABA, and are thought to inhibit responses in the vestibular center^26^. There remains some controversy regarding whether BZDs affect vestibular compensation^27^and the doses of these compounds needed to achieve benefit in vertigo are much smaller than those recommended for the treatment of anxiety^28^.

AEs not previously documented in the new labeling were identified in this study include pancreatic fibrosis. It has been demonstrated that lorazepam stimulates IL-6 production, stimulates fibrotic and inflammatory signals, promotes connective tissue proliferation and ischemic necrosis, and is associated with decreased survival in patients with pancreatic cancer^29^. Lorazepam, belonging to the class of BZDs, may be of interest in the future treatment of pancreatic cancer. BZD therapy is associated with an increased risk of motor vehicle crashes, falls, and fractures^30^. This suggests that new users may not be used to the effects of BZDs. Increased vigilance may be necessary after initiating BZDs.

The possible effects of some BZDs on the immune system have been suggested in animal models^31^but have received little attention in humans. Previous studies have hypothesized that BZDs may increase the risk of pneumonia, possibly due to nighttime and daytime sedation, increased risk of inhalation, and possible suppression of immune cells function. A population-based, propensity-matched retrospective cohort study investigated the relationship between BZDs use and the risk of chronic exacerbation pneumonia after stroke, observing that the risk of pneumonia was 2.21-fold higher in patients taking BZDs after stroke than in those not taking BZDs^32^. There is evidence suggesting an elevated risk of infections, particularly pneumonia. An increased risk of serious infections was observed across different initial BZDs types and individuals, and there was a dose-response association between cumulative doses of BZDs and risk of infection. However, the precise pathways by which BZDs influence immune function are unclear. Further research is needed to explore the neurobiological mechanisms underlying the association between BZDs use and severe infections, as it may provide safer treatment strategies for patients requiring BZDs^33^.

The time of onset for lorazepam-related AEs, as illustrated in Fig. 4, provides valuable insights into the safety of the drug. Notably, the highest frequency of AEs occurred in the first 30 days after treatment initiation, accounting for 86.10% of cases. This initial phase is critical for patient monitoring and may reflect the direct effects of the drug on the body, including pharmacokinetics and pharmacodynamics. Over time, the occurrence of AEs decreases, indicating patient adaptation to the medication, waning toxic effects or possible emergence of tolerance to the drug. Specifically, 2.6% of AEs were reported to occur between 181 and 360 days, and after one year the incidence was 5.75%, which may be related to the long-term therapeutic effect or delayed toxicity, with some individuals experiencing a later onset of AEs due to individual differences. These data emphasize the need for a comprehensive approach to pharmacovigilance and call for continued long-term follow-up of patients. Some AEs are less likely to occur but still deserve our attention.

In subgroup analyses, we performed gender and age stratification separately. In this study, we found that the risk of poisoning was more pronounced in the female population, and the AEs of sedation were more pronounced in males. It is necessary to explore sex differences in substance use outcomes and deaths because some biological differences between males and females can influence the short- and long-term effects of substance use by sex. The observed disparity in AE incidence between genders may arise from multifactorial interactions. In terms of sex hormones, such as estrogen and testosterone, they may regulate drug-metabolizing enzymes (such as CYP3A4) and receptor sensitivity, resulting in gender-specific pharmacokinetic profiles. The differences in body fat distribution and lean body mass between different genders in terms of body composition will alter the distribution volume of drugs. Gender-related healthcare-seeking behaviors may influence exposure duration and AE reporting rates. In addition, some evidence suggests that the subjective effects of drugs are influenced by ovarian hormones^34^. Interventions that provide substance use treatment and overdose response training for women in specific age groups are needed. Effective substance use treatment tools for women include safe and stable social support, positive internal self-identity, coping skills to deal with difficulties and appropriate regulation of emotions. This study suggests that we need to be aware of gender differences in clinical management. There were differences in AE across age groups, but drug abuse, sopor, and somnolence were common symptoms in patients among all three age groups. However, even short-term BZDs use in elderly patients can result in dangerous side effects. Sensitivity to BZDs is increased in the elderly^35^. In older patients, the elimination half-life of BZDs is prolonged, which can lead to drug accumulation^36^. It is essential to prevent falls in the elderly by starting with small doses to avoid overdose and toxicity. Short-term use of BZDs may be considered relatively safe when properly managed.

There are several limitations to this study. First, the FAERS database is a spontaneous reporting system that inevitably suffers from underreporting, misreporting, delayed reporting, missing information, incomplete data, and inherently introduces reporting bias and thus potential bias, which may lead to potentially biased results in the discrete analyses. Second, only AE cases were included, and the total number of patients using lorazepam is unknown, making it impossible to calculate the true incidence of associated AEs. Third, although the disproportionation analysis employed advanced signal detection algorithms such as ROR, PRR, BCPNN and MGPS, the signals of all AEs only represent statistical correlations, which can only elucidate the strength of the association between the drug and the AE, but not directly confirm causality. It cannot explain the detailed pathogenesis, and further pharmacological studies, clinical follow-up, clinical observations and prospective studies are needed to verify the existence of biological causality and further causal evaluation is needed. Finally, most reports are mainly from North America and Europe, and these findings may be restricted to specific populations, given the differences in countries, regions, and ethnicities, as well as differences in the importance attached to AE. Despite these limitations, the FAERS database remains a valuable resource and an important tool for post-marketing surveillance, and the signals identified through big data analysis for post-marketing drug surveillance remain clinically relevant in suggesting potential drug risks, and our results may provide valuable insights and references for further research.

## Conclusions

Our study conducted a systematic and comprehensive exploration of lorazepam-related signals based on the FAERS database. The AEs identified in this study were largely consistent with those previously reported, but some unexpected potential AEs such as tachycardia, rhabdomyolysis, kluver-bucy syndrome, maximal voluntary ventilation abnormal, neologism, phagophobia, pancreatic fibrosis, induced abortion failed, floppy infant, congenital pneumonia, and mitochondrial encephalomyopathy were also identified. Substantial guidance is provided for the clinical application of lorazepam in the treatment of anxiety and depression. Due to some limitations, further pharmacologic studies, prospective clinical trials are needed to validate and inform clinical decisions.

## Electronic supplementary material

Below is the link to the electronic supplementary material.

Supplementary Material 1

## Data Availability

All data is publicly available on the FDA website, the original contributions presented in the study are included in the article/Supplementary Material, further inquiries can be directed to the corresponding author.
