# Supplementary material for: A real-world pharmacovigilance study of lorazepam based on the FDA adverse event reporting system database
Source: Sci Rep. 2025 Jun 24;15:20272. doi: 10.1038/s41598-025-05680-z (PMC12187914; doi:10.1038/s41598-025-05680-z)
Supplement: Supplementary file 1 — Supplementary Material 1 [file 41598_2025_5680_MOESM1_ESM.docx]

***Supplementary Material***

**A real-world pharmacovigilance study of lorazepam based on the FDA adverse event reporting system database**

**Chunyue** **Fang^1,4#^, Xiaoyan Xu^2#^, Jianyi Li^3#^, Yuanyuan Zhong^1*^, Wei Dai^1^, Jin Wen^1^,** **Qionghui Yang^1^, Ruixiang** **Chen^1^**

^1^Department of Pharmacy, The Third People’s Hospital of Yunnan Province, Kunming, Yunnan, 650011, China

^2^Department of Ophthalmology, Southern theater general hospital, Guangzhou, 510010，China

^3^Department of Orthopaedics，The Third Affiliated Hospital，Sun Yat-sen University，Guangzhou, 510630, China

^4^College of Pharmacy, Dali University, Dali, Yunnan, 671003, China.

* **Correspondence**

Yuanyuan Zhong,

zhongy02@126.com

**^#^**These authors have contributed equally to this work.

**Supplementary Tables**

Supplementary Table S1. The top 50 signal strength of AEs of lorazepam in female ranked by the frequency at the PTs level in FAERS database.

| SOC | PTs | Case reports  (n=) | ROR  (95%CI) | PRR ( 95%CI ) | IC  (IC025) | EBGM  (EBGM05) |
| --- | --- | --- | --- | --- | --- | --- |
| psychiatric disorders | drug abuse | 1040 | 34.85(32.72, 37.11) | 33.75(31.82, 35.79) | 5.03(4.93) | 32.57(30.9) |
| psychiatric disorders | sopor | 810 | 107.98(100.32, 116.23) | 105.28(97.34, 113.87) | 6.56(6.46) | 94.41(88.77) |
| psychiatric disorders | suicide attempt | 428 | 13.42(12.19, 14.78) | 13.26(12.02, 14.63) | 3.71(3.57) | 13.08(12.07) |
| psychiatric disorders | intentional self-injury | 286 | 24.72(21.97, 27.82) | 24.51(21.79, 27.57) | 4.58(4.41) | 23.89(21.64) |
| psychiatric disorders | bradyphrenia | 142 | 38.47(32.51, 45.52) | 38.3(32.11, 45.69) | 5.2(4.96) | 36.79(31.96) |
| injury, poisoning and procedural complications | poisoning | 134 | 23(19.37, 27.31) | 22.91(19.21, 27.33) | 4.48(4.24) | 22.37(19.37) |
| psychiatric disorders | catatonia | 66 | 31.15(24.36, 39.82) | 31.08(24.09, 40.1) | 4.91(4.56) | 30.08(24.5) |
| respiratory, thoracic and mediastinal disorders | respiratory depression | 66 | 13.52(10.6, 17.25) | 13.5(10.67, 17.08) | 3.73(3.39) | 13.31(10.86) |
| nervous system disorders | bradykinesia | 58 | 22.92(17.66, 29.76) | 22.88(17.73, 29.52) | 4.48(4.11) | 22.34(17.96) |
| nervous system disorders | neuroleptic malignant syndrome | 53 | 12.87(9.81, 16.88) | 12.85(9.77, 16.91) | 3.67(3.28) | 12.69(10.11) |
| psychiatric disorders | substance abuse | 49 | 17.5(13.19, 23.22) | 17.47(13.28, 22.99) | 4.1(3.7) | 17.16(13.54) |
| eye disorders | miosis | 49 | 16.46(12.41, 21.84) | 16.44(12.49, 21.63) | 4.01(3.61) | 16.16(12.76) |
| psychiatric disorders | psychomotor retardation | 44 | 32.54(24.08, 43.96) | 32.5(24.22, 43.61) | 4.97(4.54) | 31.4(24.41) |
| nervous system disorders | slow speech | 44 | 38.22(28.26, 51.68) | 38.17(28.45, 51.22) | 5.2(4.77) | 36.66(28.48) |
| general disorders and administration site conditions | alcohol interaction | 39 | 52.41(37.95, 72.4) | 52.35(38.26, 71.63) | 5.63(5.17) | 49.54(37.81) |
| psychiatric disorders | drug use disorder | 29 | 12.95(8.97, 18.68) | 12.94(8.92, 18.78) | 3.67(3.15) | 12.77(9.39) |
| psychiatric disorders | agoraphobia | 27 | 32.64(22.23, 47.92) | 32.61(22.03, 48.26) | 4.98(4.43) | 31.51(22.85) |
| psychiatric disorders | derealisation | 25 | 22.39(15.05, 33.3) | 22.37(15.12, 33.11) | 4.45(3.89) | 21.86(15.68) |
| investigations | coma scale abnormal | 25 | 18.82(12.66, 27.96) | 18.8(12.7, 27.82) | 4.2(3.64) | 18.44(13.24) |
| nervous system disorders | psychomotor skills impaired | 24 | 21.24(14.17, 31.84) | 21.23(14.07, 32.04) | 4.38(3.8) | 20.76(14.8) |
| psychiatric disorders | depersonalisation/derealisation disorder | 23 | 31.5(20.78, 47.75) | 31.48(20.86, 47.51) | 4.93(4.34) | 30.46(21.5) |
| general disorders and administration site conditions | paradoxical drug reaction | 23 | 14.65(9.7, 22.13) | 14.64(9.7, 22.1) | 3.85(3.27) | 14.43(10.22) |
| psychiatric disorders | alcohol abuse | 21 | 22.99(14.91, 35.45) | 22.97(14.92, 35.35) | 4.49(3.88) | 22.43(15.61) |
| gastrointestinal disorders | bezoar | 21 | 34.38(22.23, 53.16) | 34.36(22.32, 52.88) | 5.05(4.44) | 33.14(23.01) |
| injury, poisoning and procedural complications | sedation complication | 20 | 16.55(10.63, 25.76) | 16.54(10.54, 25.96) | 4.02(3.4) | 16.26(11.23) |
| psychiatric disorders | somatic symptom disorder | 17 | 29.94(18.46, 48.54) | 29.92(18.33, 48.84) | 4.86(4.18) | 29(19.35) |
| psychiatric disorders | disorganised speech | 16 | 21.3(12.97, 34.97) | 21.29(13.04, 34.75) | 4.38(3.69) | 20.82(13.75) |
| respiratory, thoracic and mediastinal disorders | bradypnoea | 16 | 14.76(9.01, 24.2) | 14.76(9.04, 24.09) | 3.86(3.17) | 14.54(9.61) |
| investigations | drug screen negative | 15 | 16.7(10.02, 27.83) | 16.69(10.03, 27.78) | 4.04(3.32) | 16.41(10.7) |
| musculoskeletal and connective tissue disorders | sacroiliitis | 15 | 14.54(8.73, 24.21) | 14.53(8.73, 24.19) | 3.84(3.13) | 14.32(9.34) |
| nervous system disorders | hyperammonaemic encephalopathy | 13 | 19.13(11.04, 33.14) | 19.12(11.04, 33.1) | 4.23(3.46) | 18.75(11.84) |
| immune system disorders | sensitisation | 13 | 22.85(13.17, 39.62) | 22.84(13.19, 39.54) | 4.48(3.71) | 22.3(14.07) |
| psychiatric disorders | mutism | 11 | 13.4(7.39, 24.3) | 13.39(7.44, 24.11) | 3.72(2.9) | 13.21(8.03) |
| gastrointestinal disorders | rectal polyp | 11 | 23.48(12.9, 42.73) | 23.47(12.78, 43.09) | 4.52(3.69) | 22.9(13.88) |
| investigations | anion gap increased | 9 | 14.24(7.37, 27.51) | 14.24(7.31, 27.73) | 3.81(2.91) | 14.03(8.09) |
| investigations | false negative investigation result | 9 | 43.79(22.43, 85.49) | 43.78(22.48, 85.25) | 5.39(4.47) | 41.8(23.88) |
| musculoskeletal and connective tissue disorders | hypotonia neonatal | 9 | 14.49(7.5, 28) | 14.49(7.44, 28.22) | 3.84(2.93) | 14.28(8.23) |
| psychiatric disorders | intrusive thoughts | 8 | 18.14(9.01, 36.54) | 18.14(8.96, 36.73) | 4.15(3.2) | 17.8(9.91) |
| investigations | gastric ph decreased | 8 | 12.44(6.19, 24.99) | 12.44(6.14, 25.19) | 3.62(2.67) | 12.28(6.85) |
| infections and infestations | sputum purulent | 8 | 25.22(12.49, 50.93) | 25.22(12.45, 51.07) | 4.62(3.66) | 24.56(13.64) |
| immune system disorders | secondary immunodeficiency | 8 | 19.73(9.79, 39.75) | 19.72(9.74, 39.93) | 4.27(3.32) | 19.32(10.75) |
| nervous system disorders | cogwheel rigidity | 7 | 13.86(6.57, 29.24) | 13.86(6.58, 29.19) | 3.77(2.76) | 13.66(7.32) |
| investigations | protein c increased | 7 | 143.96(64.84, 319.64) | 143.93(64.44, 321.48) | 6.96(5.88) | 124.31(63.78) |
| investigations | osmolar gap increased | 7 | 140.76(63.48, 312.16) | 140.73(63.01, 314.33) | 6.93(5.86) | 121.92(62.61) |
| respiratory, thoracic and mediastinal disorders | neonatal asphyxia | 7 | 29.19(13.75, 61.97) | 29.18(13.86, 61.45) | 4.82(3.81) | 28.3(15.08) |
| respiratory, thoracic and mediastinal disorders | irregular breathing | 7 | 25.24(11.91, 53.49) | 25.23(11.98, 53.13) | 4.62(3.6) | 24.57(13.11) |
| injury, poisoning and procedural complications | induced abortion failed | 7 | 57.59(26.82, 123.64) | 57.57(26.81, 123.64) | 5.76(4.73) | 54.19(28.59) |
| cardiac disorders | myocardial fibrosis | 7 | 21.99(10.39, 46.55) | 21.99(10.44, 46.31) | 4.43(3.41) | 21.49(11.48) |
| psychiatric disorders | withdrawal catatonia | 6 | 89.01(38.48, 205.89) | 88.99(38.31, 206.71) | 6.34(5.22) | 81.11(40.21) |
| psychiatric disorders | echolalia | 6 | 60.33(26.4, 137.86) | 60.31(26.48, 137.37) | 5.82(4.72) | 56.61(28.35) |

ROR, reporting odds ratio; CI, confidence interval; PRR, proportional reporting ratio; IC, information component; EBGM, empirical Bayesian geometric mean; IC025, the lower limit of 95% CI of the IC; EBGM05, the lower limit of 95% CI of EBGM; PT: preferred term.

Supplementary Table S2. The top 50 signal strength of AEs of lorazepam in male ranked by the frequency at the PTs level in FAERS database.

| SOC | PTs | Case reports  (n=) | ROR  (95%CI) | PRR ( 95%CI ) | IC  (IC025) | EBGM  (EBGM05) |
| --- | --- | --- | --- | --- | --- | --- |
| psychiatric disorders | drug abuse | 662 | 18.63(17.23, 20.15) | 17.95(16.6, 19.41) | 4.14(4.03) | 17.66(16.54) |
| psychiatric disorders | sopor | 359 | 99.03(88.77, 110.47) | 96.99(87.94, 106.98) | 6.47(6.31) | 88.6(80.86) |
| psychiatric disorders | suicide attempt | 197 | 10.58(9.19, 12.18) | 10.47(9.13, 12.01) | 3.37(3.17) | 10.37(9.22) |
| psychiatric disorders | intentional self-injury | 155 | 27.37(23.32, 32.13) | 27.14(23.2, 31.75) | 4.73(4.5) | 26.45(23.13) |
| psychiatric disorders | delirium | 144 | 10.75(9.12, 12.68) | 10.67(9.12, 12.48) | 3.4(3.16) | 10.57(9.21) |
| nervous system disorders | sedation | 131 | 15.6(13.12, 18.56) | 15.49(12.98, 18.48) | 3.93(3.68) | 15.28(13.21) |
| psychiatric disorders | bradyphrenia | 84 | 41.19(33.1, 51.26) | 40.99(33.04, 50.85) | 5.3(4.99) | 39.44(32.84) |
| respiratory, thoracic and mediastinal disorders | respiratory depression | 75 | 15.06(11.98, 18.93) | 15(11.86, 18.98) | 3.89(3.56) | 14.79(12.22) |
| injury, poisoning and procedural complications | poisoning | 74 | 15.39(12.22, 19.37) | 15.33(12.12, 19.39) | 3.92(3.59) | 15.11(12.47) |
| nervous system disorders | neuroleptic malignant syndrome | 65 | 11.69(9.15, 14.93) | 11.65(9.21, 14.74) | 3.53(3.18) | 11.53(9.39) |
| psychiatric disorders | catatonia | 52 | 26.47(20.09, 34.87) | 26.39(20.06, 34.72) | 4.69(4.29) | 25.75(20.44) |
| nervous system disorders | hypokinesia | 40 | 8.99(6.58, 12.27) | 8.97(6.56, 12.27) | 3.15(2.71) | 8.9(6.86) |
| eye disorders | miosis | 35 | 10.49(7.52, 14.64) | 10.47(7.5, 14.61) | 3.38(2.9) | 10.38(7.85) |
| nervous system disorders | bradykinesia | 34 | 17.35(12.36, 24.36) | 17.32(12.41, 24.17) | 4.09(3.61) | 17.04(12.83) |
| nervous system disorders | stupor | 24 | 19.59(13.07, 29.34) | 19.56(12.96, 29.52) | 4.26(3.69) | 19.21(13.7) |
| general disorders and administration site conditions | paradoxical drug reaction | 23 | 19.01(12.58, 28.73) | 18.99(12.58, 28.66) | 4.22(3.64) | 18.66(13.21) |
| injury, poisoning and procedural complications | sedation complication | 22 | 35.2(23, 53.86) | 35.16(22.84, 54.11) | 5.09(4.49) | 34.01(23.83) |
| psychiatric disorders | withdrawal catatonia | 18 | 372.55(217.02, 639.57) | 372.17(214.98, 644.29) | 8.09(7.36) | 272.45(173.34) |
| psychiatric disorders | psychomotor retardation | 16 | 16.05(9.79, 26.3) | 16.03(9.82, 26.17) | 3.98(3.29) | 15.8(10.45) |
| respiratory, thoracic and mediastinal disorders | hypercapnia | 15 | 13.4(8.05, 22.31) | 13.39(8.04, 22.29) | 3.73(3.01) | 13.23(8.64) |
| gastrointestinal disorders | megacolon | 15 | 23.95(14.35, 39.98) | 23.93(14.38, 39.83) | 4.55(3.83) | 23.4(15.25) |
| investigations | drug screen negative | 13 | 22.57(13.02, 39.11) | 22.55(13.03, 39.04) | 4.46(3.7) | 22.08(13.94) |
| psychiatric disorders | self-injurious ideation | 12 | 9.66(5.47, 17.06) | 9.66(5.47, 17.05) | 3.26(2.47) | 9.57(5.95) |
| nervous system disorders | drop attacks | 12 | 59.35(33.15, 106.24) | 59.3(32.94, 106.76) | 5.81(5) | 56.08(34.45) |
| nervous system disorders | drug withdrawal convulsions | 12 | 18.21(10.29, 32.24) | 18.2(10.31, 32.13) | 4.16(3.37) | 17.9(11.1) |
| psychiatric disorders | persecutory delusion | 11 | 11.13(6.14, 20.16) | 11.12(6.18, 20.02) | 3.46(2.64) | 11.01(6.7) |
| psychiatric disorders | disinhibition | 10 | 12.39(6.64, 23.12) | 12.39(6.62, 23.2) | 3.61(2.76) | 12.25(7.27) |
| psychiatric disorders | staring | 10 | 10.03(5.38, 18.69) | 10.02(5.35, 18.76) | 3.31(2.45) | 9.93(5.9) |
| psychiatric disorders | panic disorder | 9 | 8.94(4.64, 17.24) | 8.94(4.68, 17.07) | 3.15(2.25) | 8.87(5.12) |
| psychiatric disorders | delirium tremens | 8 | 32.96(16.3, 66.66) | 32.95(16.27, 66.73) | 5(4.04) | 31.94(17.72) |
| psychiatric disorders | adjustment disorder | 8 | 23.17(11.49, 46.7) | 23.16(11.44, 46.9) | 4.5(3.55) | 22.66(12.61) |
| psychiatric disorders | negativism | 8 | 21.62(10.73, 43.56) | 21.61(10.67, 43.76) | 4.4(3.45) | 21.18(11.79) |
| general disorders and administration site conditions | alcohol interaction | 8 | 9.21(4.59, 18.49) | 9.21(4.55, 18.65) | 3.19(2.24) | 9.14(5.1) |
| psychiatric disorders | agoraphobia | 7 | 18.97(8.98, 40.07) | 18.96(9, 39.93) | 4.22(3.21) | 18.63(9.97) |
| psychiatric disorders | daydreaming | 7 | 13.09(6.21, 27.59) | 13.08(6.21, 27.55) | 3.69(2.69) | 12.93(6.93) |
| psychiatric disorders | psychotic symptom | 7 | 9.38(4.46, 19.75) | 9.38(4.45, 19.75) | 3.22(2.21) | 9.3(4.99) |
| nervous system disorders | sleep deficit | 7 | 12.4(5.89, 26.14) | 12.4(5.89, 26.11) | 3.62(2.61) | 12.26(6.57) |
| nervous system disorders | hyporeflexia | 7 | 9.3(4.42, 19.57) | 9.29(4.41, 19.56) | 3.2(2.2) | 9.22(4.95) |
| investigations | electrocardiogram repolarisation abnormality | 7 | 23.34(11.03, 49.38) | 23.33(11.08, 49.13) | 4.51(3.5) | 22.83(12.19) |
| psychiatric disorders | confabulation | 6 | 65.38(28.63, 149.31) | 65.36(28.69, 148.88) | 5.94(4.84) | 61.46(30.8) |
| psychiatric disorders | poverty of speech | 6 | 44.71(19.74, 101.29) | 44.7(19.62, 101.82) | 5.42(4.33) | 42.85(21.62) |
| psychiatric disorders | derealisation | 6 | 9.74(4.36, 21.78) | 9.74(4.36, 21.75) | 3.27(2.2) | 9.66(4.93) |
| psychiatric disorders | delusion of grandeur | 6 | 24.42(10.86, 54.89) | 24.41(10.93, 54.52) | 4.58(3.49) | 23.86(12.12) |
| psychiatric disorders | illusion | 6 | 9.21(4.12, 20.58) | 9.21(4.12, 20.57) | 3.19(2.12) | 9.14(4.66) |
| metabolism and nutrition disorders | hyperosmolar state | 6 | 45.38(20.03, 102.83) | 45.36(19.91, 103.32) | 5.44(4.35) | 43.46(21.92) |
| cardiac disorders | myocardial fibrosis | 6 | 16(7.14, 35.85) | 16(7.16, 35.74) | 3.98(2.9) | 15.76(8.03) |
| psychiatric disorders | depersonalisation/derealisation disorder | 5 | 12.7(5.26, 30.68) | 12.7(5.26, 30.68) | 3.65(2.49) | 12.55(6) |
| psychiatric disorders | mutism | 5 | 11.36(4.7, 27.43) | 11.36(4.7, 27.44) | 3.49(2.33) | 11.24(5.38) |
| psychiatric disorders | malignant catatonia | 5 | 41.53(16.98, 101.59) | 41.52(16.85, 102.29) | 5.32(4.14) | 39.93(18.89) |
| psychiatric disorders | soliloquy | 5 | 14.9(6.16, 36.04) | 14.9(6.17, 35.99) | 3.88(2.71) | 14.7(7.02) |

ROR, reporting odds ratio; CI, confidence interval; PRR, proportional reporting ratio; IC, information component; EBGM, empirical Bayesian geometric mean; IC025, the lower limit of 95% CI of the IC; EBGM05, the lower limit of 95% CI of EBGM; PT: preferred term.

Supplementary Table S3.The top 50 signal strength of AEs of lorazepam ranked by the frequency at the PTs level in FAERS database(age<=18).

| SOC | PTs | Case reports  (n=) | ROR  (95%CI) | PRR ( 95%CI ) | IC  (IC025) | EBGM  (EBGM05) |
| --- | --- | --- | --- | --- | --- | --- |
| psychiatric disorders | drug abuse | 65 | 17.85(13.91, 22.9) | 17.41(13.76, 22.03) | 4.09(3.73) | 17.04(13.83) |
| psychiatric disorders | sopor | 49 | 73.38(54.59, 98.62) | 71.94(53.62, 96.53) | 6.04(5.62) | 65.79(51.37) |
| nervous system disorders | somnolence | 43 | 3.15(2.33, 4.26) | 3.11(2.32, 4.17) | 1.63(1.2) | 3.1(2.41) |
| psychiatric disorders | agitation | 38 | 4.96(3.6, 6.84) | 4.9(3.58, 6.7) | 2.28(1.83) | 4.87(3.72) |
| psychiatric disorders | suicide attempt | 33 | 4.55(3.23, 6.43) | 4.51(3.23, 6.29) | 2.17(1.68) | 4.49(3.36) |
| injury, poisoning and procedural complications | intentional overdose | 32 | 3.56(2.51, 5.05) | 3.53(2.48, 5.02) | 1.81(1.32) | 3.52(2.62) |
| psychiatric disorders | intentional self-injury | 31 | 8.9(6.23, 12.71) | 8.8(6.18, 12.52) | 3.12(2.62) | 8.71(6.46) |
| injury, poisoning and procedural complications | accidental exposure to product by child | 31 | 4.69(3.29, 6.69) | 4.64(3.26, 6.6) | 2.21(1.7) | 4.62(3.43) |
| psychiatric disorders | confusional state | 28 | 7.27(5, 10.58) | 7.2(4.96, 10.45) | 2.84(2.31) | 7.14(5.22) |
| general disorders and administration site conditions | drug ineffective for unapproved indication | 28 | 8.35(5.74, 12.15) | 8.27(5.7, 12) | 3.03(2.5) | 8.19(5.99) |
| general disorders and administration site conditions | gait disturbance | 25 | 6.73(4.53, 9.99) | 6.67(4.51, 9.87) | 2.73(2.17) | 6.62(4.75) |
| psychiatric disorders | hallucination | 24 | 5.35(3.57, 8.01) | 5.31(3.59, 7.86) | 2.4(1.83) | 5.28(3.76) |
| injury, poisoning and procedural complications | accidental overdose | 24 | 3.9(2.61, 5.84) | 3.88(2.62, 5.74) | 1.95(1.38) | 3.86(2.76) |
| general disorders and administration site conditions | asthenia | 19 | 3.38(2.15, 5.31) | 3.36(2.14, 5.27) | 1.74(1.11) | 3.35(2.3) |
| nervous system disorders | psychomotor hyperactivity | 17 | 4.27(2.65, 6.89) | 4.25(2.66, 6.8) | 2.08(1.41) | 4.23(2.83) |
| injury, poisoning and procedural complications | medication error | 17 | 4.02(2.49, 6.48) | 4(2.5, 6.4) | 1.99(1.32) | 3.98(2.67) |
| injury, poisoning and procedural complications | intentional product misuse | 17 | 5.7(3.53, 9.21) | 5.67(3.54, 9.08) | 2.49(1.82) | 5.64(3.78) |
| respiratory, thoracic and mediastinal disorders | respiratory depression | 17 | 11.68(7.22, 18.88) | 11.6(7.25, 18.57) | 3.52(2.84) | 11.44(7.65) |
| nervous system disorders | depressed level of consciousness | 16 | 4.64(2.84, 7.6) | 4.62(2.83, 7.54) | 2.2(1.51) | 4.6(3.04) |
| nervous system disorders | neuroleptic malignant syndrome | 16 | 21.14(12.84, 34.79) | 21.01(12.87, 34.29) | 4.36(3.66) | 20.47(13.49) |
| respiratory, thoracic and mediastinal disorders | hypoxia | 16 | 5.81(3.55, 9.51) | 5.78(3.54, 9.43) | 2.52(1.83) | 5.74(3.8) |
| eye disorders | mydriasis | 15 | 6.01(3.61, 10.01) | 5.98(3.59, 9.95) | 2.57(1.86) | 5.94(3.88) |
| nervous system disorders | speech disorder | 13 | 6.08(3.52, 10.51) | 6.05(3.49, 10.47) | 2.59(1.83) | 6.01(3.8) |
| general disorders and administration site conditions | withdrawal syndrome | 13 | 10.21(5.9, 17.68) | 10.16(5.87, 17.59) | 3.33(2.56) | 10.04(6.35) |
| psychiatric disorders | delirium | 12 | 8.29(4.68, 14.65) | 8.25(4.67, 14.56) | 3.03(2.24) | 8.17(5.07) |
| nervous system disorders | sedation | 12 | 8.34(4.71, 14.75) | 8.3(4.7, 14.65) | 3.04(2.25) | 8.22(5.1) |
| nervous system disorders | aphasia | 12 | 10.81(6.1, 19.13) | 10.76(6.09, 19) | 3.41(2.62) | 10.62(6.59) |
| nervous system disorders | dysarthria | 11 | 6.27(3.46, 11.37) | 6.25(3.47, 11.25) | 2.63(1.81) | 6.2(3.77) |
| respiratory, thoracic and mediastinal disorders | respiratory arrest | 11 | 4.27(2.36, 7.73) | 4.25(2.36, 7.65) | 2.08(1.26) | 4.24(2.58) |
| psychiatric disorders | psychotic disorder | 10 | 5.47(2.93, 10.21) | 5.45(2.91, 10.2) | 2.44(1.58) | 5.42(3.22) |
| psychiatric disorders | drug dependence | 10 | 6.05(3.24, 11.28) | 6.03(3.22, 11.29) | 2.58(1.72) | 5.99(3.55) |
| nervous system disorders | balance disorder | 10 | 9.05(4.85, 16.91) | 9.02(4.82, 16.89) | 3.16(2.3) | 8.92(5.29) |
| investigations | blood creatine phosphokinase increased | 10 | 6.17(3.3, 11.5) | 6.14(3.28, 11.5) | 2.61(1.75) | 6.1(3.62) |
| respiratory, thoracic and mediastinal disorders | apnoea | 10 | 4.81(2.58, 8.97) | 4.8(2.56, 8.99) | 2.26(1.4) | 4.77(2.83) |
| general disorders and administration site conditions | therapy non-responder | 10 | 4.31(2.31, 8.04) | 4.3(2.3, 8.05) | 2.1(1.24) | 4.28(2.54) |
| psychiatric disorders | catatonia | 9 | 16.85(8.7, 32.66) | 16.79(8.62, 32.69) | 4.04(3.13) | 16.45(9.46) |
| psychiatric disorders | substance abuse | 9 | 23.92(12.31, 46.51) | 23.84(12.24, 46.42) | 4.53(3.62) | 23.14(13.27) |
| nervous system disorders | slow speech | 9 | 62.21(31.49, 122.88) | 61.99(31.22, 123.1) | 5.84(4.91) | 57.37(32.46) |
| nervous system disorders | cognitive disorder | 9 | 7.1(3.68, 13.71) | 7.08(3.71, 13.52) | 2.81(1.91) | 7.02(4.05) |
| nervous system disorders | amnesia | 9 | 7.87(4.08, 15.2) | 7.85(4.11, 14.99) | 2.96(2.06) | 7.78(4.48) |
| musculoskeletal and connective tissue disorders | hypotonia neonatal | 9 | 10.77(5.57, 20.83) | 10.74(5.52, 20.91) | 3.41(2.5) | 10.6(6.11) |
| psychiatric disorders | psychomotor retardation | 8 | 27.51(13.57, 55.76) | 27.43(13.55, 55.55) | 4.73(3.77) | 26.5(14.67) |
| nervous system disorders | ataxia | 8 | 5.32(2.65, 10.67) | 5.3(2.67, 10.52) | 2.4(1.45) | 5.27(2.94) |
| psychiatric disorders | hallucination, auditory | 7 | 4.71(2.24, 9.91) | 4.7(2.23, 9.9) | 2.22(1.22) | 4.67(2.51) |
| nervous system disorders | mental impairment | 7 | 11.74(5.56, 24.79) | 11.71(5.56, 24.66) | 3.53(2.52) | 11.54(6.18) |
| respiratory, thoracic and mediastinal disorders | respiratory acidosis | 7 | 12.45(5.89, 26.31) | 12.42(5.9, 26.16) | 3.61(2.6) | 12.24(6.54) |
| musculoskeletal and connective tissue disorders | muscle rigidity | 7 | 8.13(3.86, 17.14) | 8.11(3.85, 17.08) | 3.01(2) | 8.03(4.3) |
| blood and lymphatic system disorders | eosinophilia | 7 | 7.16(3.4, 15.08) | 7.14(3.39, 15.04) | 2.82(1.82) | 7.08(3.79) |
| psychiatric disorders | delusion | 6 | 10.95(4.89, 24.54) | 10.93(4.89, 24.41) | 3.43(2.35) | 10.79(5.49) |
| psychiatric disorders | disorganised speech | 6 | 68.01(29.47, 156.93) | 67.85(29.21, 157.61) | 5.96(4.84) | 62.35(30.97) |

ROR, reporting odds ratio; CI, confidence interval; PRR, proportional reporting ratio; IC, information component; EBGM, empirical Bayesian geometric mean; IC025, the lower limit of 95% CI of the IC; EBGM05, the lower limit of 95% CI of EBGM; PT: preferred term.

Supplementary Table S4.The top 50 signal strength of AEs of lorazepam ranked by the frequency at the PTs level in FAERS database(age=18~65).

| SOC | PTs | Case reports  (n=) | ROR  (95%CI) | PRR ( 95%CI ) | IC  (IC025) | EBGM  (EBGM05) |
| --- | --- | --- | --- | --- | --- | --- |
| psychiatric disorders | drug abuse | 1297 | 20.79(19.65, 22) | 19.83(18.7, 21.03) | 4.27(4.19) | 19.34(18.45) |
| psychiatric disorders | sopor | 710 | 86.24(79.72, 93.28) | 83.97(77.64, 90.82) | 6.24(6.13) | 75.54(70.73) |
| psychiatric disorders | completed suicide | 556 | 7.81(7.18, 8.5) | 7.67(7.09, 8.3) | 2.93(2.8) | 7.6(7.08) |
| injury, poisoning and procedural complications | toxicity to various agents | 428 | 4.51(4.09, 4.96) | 4.45(4.03, 4.91) | 2.15(2.01) | 4.43(4.09) |
| nervous system disorders | somnolence | 403 | 4.5(4.08, 4.97) | 4.45(4.03, 4.91) | 2.15(2) | 4.43(4.07) |
| psychiatric disorders | suicide attempt | 392 | 10.03(9.08, 11.09) | 9.9(8.98, 10.92) | 3.29(3.15) | 9.78(9) |
| injury, poisoning and procedural complications | intentional overdose | 363 | 8.95(8.06, 9.93) | 8.84(8.01, 9.75) | 3.13(2.98) | 8.75(8.02) |
| psychiatric disorders | intentional self-injury | 344 | 27.06(24.28, 30.15) | 26.72(24.23, 29.47) | 4.69(4.54) | 25.83(23.59) |
| injury, poisoning and procedural complications | overdose | 339 | 4.34(3.9, 4.83) | 4.3(3.9, 4.74) | 2.1(1.94) | 4.28(3.91) |
| psychiatric disorders | drug dependence | 236 | 9.05(7.95, 10.29) | 8.98(7.83, 10.3) | 3.15(2.97) | 8.88(7.97) |
| psychiatric disorders | agitation | 229 | 5.95(5.22, 6.78) | 5.91(5.15, 6.78) | 2.55(2.37) | 5.87(5.26) |
| injury, poisoning and procedural complications | poisoning | 196 | 19.58(16.98, 22.57) | 19.44(16.95, 22.3) | 4.25(4.04) | 18.97(16.84) |
| cardiac disorders | tachycardia | 196 | 3.75(3.26, 4.32) | 3.73(3.25, 4.28) | 1.89(1.69) | 3.71(3.3) |
| general disorders and administration site conditions | withdrawal syndrome | 184 | 9.12(7.89, 10.56) | 9.07(7.91, 10.4) | 3.17(2.96) | 8.97(7.94) |
| nervous system disorders | coma | 178 | 6.15(5.3, 7.13) | 6.11(5.22, 7.15) | 2.6(2.39) | 6.07(5.36) |
| cardiac disorders | cardiac arrest | 175 | 3.65(3.15, 4.24) | 3.64(3.11, 4.26) | 1.86(1.64) | 3.62(3.2) |
| general disorders and administration site conditions | drug withdrawal syndrome | 173 | 6.02(5.18, 7) | 5.99(5.12, 7.01) | 2.57(2.36) | 5.95(5.25) |
| psychiatric disorders | suicidal ideation | 167 | 3.13(2.69, 3.64) | 3.12(2.67, 3.65) | 1.64(1.42) | 3.11(2.73) |
| psychiatric disorders | bradyphrenia | 162 | 42.23(36.03, 49.49) | 41.97(35.88, 49.09) | 5.31(5.09) | 39.78(34.83) |
| respiratory, thoracic and mediastinal disorders | respiratory arrest | 156 | 7.75(6.61, 9.07) | 7.71(6.59, 9.02) | 2.93(2.71) | 7.64(6.69) |
| nervous system disorders | sedation | 140 | 10.88(9.2, 12.86) | 10.83(9.26, 12.67) | 3.42(3.18) | 10.69(9.29) |
| psychiatric disorders | delirium | 137 | 10.89(9.2, 12.9) | 10.84(9.09, 12.93) | 3.42(3.18) | 10.7(9.29) |
| nervous system disorders | depressed level of consciousness | 128 | 6.07(5.1, 7.22) | 6.04(5.06, 7.21) | 2.59(2.34) | 6(5.19) |
| psychiatric disorders | aggression | 108 | 4.49(3.71, 5.42) | 4.47(3.67, 5.44) | 2.15(1.88) | 4.45(3.8) |
| nervous system disorders | speech disorder | 107 | 4.3(3.55, 5.2) | 4.28(3.52, 5.21) | 2.09(1.82) | 4.26(3.64) |
| nervous system disorders | dysarthria | 97 | 4.65(3.81, 5.68) | 4.64(3.81, 5.64) | 2.21(1.92) | 4.62(3.9) |
| general disorders and administration site conditions | drug ineffective for unapproved indication | 92 | 4.22(3.44, 5.19) | 4.21(3.46, 5.12) | 2.07(1.77) | 4.19(3.53) |
| cardiac disorders | cardio-respiratory arrest | 91 | 3.37(2.74, 4.14) | 3.36(2.76, 4.09) | 1.74(1.45) | 3.35(2.82) |
| psychiatric disorders | disorientation | 88 | 4.52(3.67, 5.58) | 4.51(3.64, 5.6) | 2.17(1.87) | 4.49(3.77) |
| psychiatric disorders | catatonia | 85 | 28.96(23.31, 35.98) | 28.87(23.27, 35.82) | 4.8(4.49) | 27.83(23.21) |
| nervous system disorders | neuroleptic malignant syndrome | 85 | 10.71(8.64, 13.27) | 10.68(8.61, 13.25) | 3.4(3.09) | 10.54(8.81) |
| psychiatric disorders | psychotic disorder | 84 | 4.74(3.82, 5.87) | 4.73(3.81, 5.87) | 2.23(1.93) | 4.7(3.93) |
| respiratory, thoracic and mediastinal disorders | respiratory depression | 79 | 10.88(8.71, 13.58) | 10.85(8.75, 13.46) | 3.42(3.1) | 10.7(8.89) |
| psychiatric disorders | hallucination | 78 | 3.17(2.54, 3.96) | 3.17(2.56, 3.93) | 1.66(1.34) | 3.16(2.62) |
| psychiatric disorders | panic attack | 78 | 3.34(2.67, 4.17) | 3.33(2.68, 4.13) | 1.73(1.41) | 3.32(2.76) |
| musculoskeletal and connective tissue disorders | rhabdomyolysis | 76 | 3.51(2.8, 4.4) | 3.5(2.82, 4.34) | 1.8(1.48) | 3.49(2.89) |
| nervous system disorders | unresponsive to stimuli | 75 | 5.71(4.54, 7.16) | 5.69(4.5, 7.2) | 2.5(2.17) | 5.66(4.68) |
| nervous system disorders | hypokinesia | 74 | 10.58(8.41, 13.31) | 10.55(8.34, 13.35) | 3.38(3.05) | 10.42(8.6) |
| psychiatric disorders | restlessness | 71 | 3.6(2.85, 4.54) | 3.59(2.84, 4.54) | 1.84(1.5) | 3.58(2.94) |
| metabolism and nutrition disorders | metabolic acidosis | 68 | 4.03(3.17, 5.11) | 4.02(3.18, 5.09) | 2(1.66) | 4(3.28) |
| nervous system disorders | psychomotor hyperactivity | 61 | 8.4(6.53, 10.82) | 8.38(6.5, 10.81) | 3.05(2.69) | 8.3(6.72) |
| nervous system disorders | bradykinesia | 60 | 30.23(23.35, 39.15) | 30.17(23.38, 38.93) | 4.86(4.49) | 29.03(23.38) |
| eye disorders | mydriasis | 59 | 7.68(5.94, 9.93) | 7.66(5.94, 9.88) | 2.93(2.56) | 7.6(6.13) |
| psychiatric disorders | fear | 56 | 3.88(2.98, 5.04) | 3.87(3, 4.99) | 1.95(1.57) | 3.85(3.09) |
| psychiatric disorders | abnormal behaviour | 50 | 3.01(2.28, 3.98) | 3.01(2.29, 3.96) | 1.59(1.19) | 3(2.38) |
| psychiatric disorders | mental status changes | 49 | 3.64(2.75, 4.82) | 3.64(2.77, 4.79) | 1.86(1.46) | 3.63(2.87) |
| cardiac disorders | sinus tachycardia | 49 | 4.94(3.73, 6.54) | 4.93(3.75, 6.49) | 2.29(1.89) | 4.9(3.87) |
| psychiatric disorders | substance abuse | 47 | 8.53(6.4, 11.38) | 8.52(6.35, 11.43) | 3.08(2.67) | 8.43(6.63) |
| infections and infestations | pneumonia aspiration | 47 | 4.81(3.61, 6.41) | 4.81(3.58, 6.45) | 2.26(1.85) | 4.78(3.76) |
| general disorders and administration site conditions | hypothermia | 45 | 9.16(6.83, 12.3) | 9.15(6.82, 12.28) | 3.18(2.76) | 9.05(7.07) |

ROR, reporting odds ratio; CI, confidence interval; PRR, proportional reporting ratio; IC, information component; EBGM, empirical Bayesian geometric mean; IC025, the lower limit of 95% CI of the IC; EBGM05, the lower limit of 95% CI of EBGM; PT: preferred term.

Supplementary Table S5.The top 50 signal strength of AEs of lorazepam ranked by the frequency at the PTs level in FAERS database(age>=65).

| SOC | PTs | Case reports  (n=) | ROR  (95%CI) | PRR ( 95%CI ) | IC  (IC025) | EBGM  (EBGM05) |
| --- | --- | --- | --- | --- | --- | --- |
| psychiatric disorders | sopor | 347 | 99.49(88.96, 111.26) | 96.59(85.87, 108.64) | 6.46(6.3) | 87.92(80.07) |
| psychiatric disorders | drug abuse | 290 | 88.59(78.46, 100.04) | 86.44(76.85, 97.23) | 6.31(6.14) | 79.44(71.76) |
| psychiatric disorders | confusional state | 237 | 4.7(4.13, 5.35) | 4.63(4.12, 5.21) | 2.21(2.02) | 4.61(4.14) |
| nervous system disorders | somnolence | 196 | 5.05(4.39, 5.82) | 4.99(4.35, 5.72) | 2.31(2.11) | 4.97(4.41) |
| psychiatric disorders | completed suicide | 170 | 15.19(13.04, 17.69) | 14.99(12.81, 17.53) | 3.88(3.67) | 14.77(13) |
| psychiatric disorders | anxiety | 135 | 4.37(3.68, 5.18) | 4.33(3.63, 5.17) | 2.11(1.86) | 4.31(3.74) |
| injury, poisoning and procedural complications | overdose | 130 | 6.84(5.75, 8.13) | 6.77(5.68, 8.08) | 2.75(2.5) | 6.73(5.82) |
| psychiatric disorders | agitation | 124 | 10.19(8.53, 12.17) | 10.09(8.46, 12.04) | 3.32(3.07) | 10(8.61) |
| psychiatric disorders | delirium | 107 | 8.65(7.14, 10.47) | 8.58(7.05, 10.44) | 3.09(2.82) | 8.51(7.25) |
| injury, poisoning and procedural complications | toxicity to various agents | 100 | 4.38(3.6, 5.34) | 4.35(3.58, 5.29) | 2.12(1.83) | 4.34(3.68) |
| nervous system disorders | loss of consciousness | 95 | 3(2.45, 3.67) | 2.98(2.45, 3.63) | 1.57(1.28) | 2.98(2.51) |
| psychiatric disorders | drug dependence | 91 | 25.65(20.81, 31.6) | 25.46(20.52, 31.59) | 4.63(4.33) | 24.83(20.85) |
| nervous system disorders | depressed level of consciousness | 86 | 7.76(6.27, 9.6) | 7.71(6.21, 9.57) | 2.94(2.63) | 7.65(6.41) |
| nervous system disorders | sedation | 76 | 19.9(15.84, 24.99) | 19.77(15.63, 25.01) | 4.28(3.95) | 19.4(16.03) |
| psychiatric disorders | disorientation | 73 | 5.99(4.75, 7.54) | 5.96(4.71, 7.54) | 2.57(2.24) | 5.93(4.89) |
| general disorders and administration site conditions | withdrawal syndrome | 69 | 19.98(15.73, 25.38) | 19.87(15.71, 25.14) | 4.28(3.94) | 19.49(15.96) |
| psychiatric disorders | suicide attempt | 66 | 20.19(15.81, 25.78) | 20.08(15.87, 25.4) | 4.3(3.95) | 19.69(16.05) |
| nervous system disorders | coma | 65 | 6.86(5.37, 8.76) | 6.83(5.4, 8.64) | 2.76(2.41) | 6.79(5.53) |
| psychiatric disorders | hallucination | 64 | 3.06(2.39, 3.91) | 3.04(2.4, 3.85) | 1.6(1.25) | 3.04(2.47) |
| nervous system disorders | speech disorder | 59 | 4.94(3.82, 6.39) | 4.92(3.81, 6.35) | 2.29(1.93) | 4.9(3.96) |
| nervous system disorders | cognitive disorder | 54 | 5.65(4.32, 7.38) | 5.63(4.28, 7.41) | 2.49(2.1) | 5.6(4.47) |
| psychiatric disorders | intentional self-injury | 52 | 63.36(47.84, 83.93) | 63.09(47.95, 83.01) | 5.89(5.49) | 59.29(46.86) |
| cardiac disorders | tachycardia | 49 | 3.14(2.37, 4.16) | 3.13(2.38, 4.12) | 1.64(1.24) | 3.13(2.47) |
| injury, poisoning and procedural complications | medication error | 46 | 4.22(3.16, 5.64) | 4.21(3.14, 5.65) | 2.07(1.65) | 4.19(3.29) |
| psychiatric disorders | bradyphrenia | 44 | 30.21(22.37, 40.81) | 30.11(22.44, 40.4) | 4.87(4.44) | 29.23(22.73) |
| injury, poisoning and procedural complications | intentional overdose | 44 | 12.06(8.95, 16.25) | 12.02(8.96, 16.13) | 3.57(3.15) | 11.88(9.26) |
| general disorders and administration site conditions | drug withdrawal syndrome | 43 | 13.45(9.95, 18.18) | 13.4(9.99, 17.98) | 3.73(3.3) | 13.23(10.28) |
| general disorders and administration site conditions | drug ineffective for unapproved indication | 42 | 4.73(3.49, 6.41) | 4.72(3.52, 6.33) | 2.23(1.8) | 4.7(3.65) |
| respiratory, thoracic and mediastinal disorders | respiratory arrest | 39 | 7.64(5.57, 10.47) | 7.61(5.56, 10.41) | 2.92(2.47) | 7.56(5.81) |
| psychiatric disorders | mental status changes | 38 | 4.76(3.46, 6.55) | 4.75(3.47, 6.5) | 2.24(1.79) | 4.73(3.62) |
| nervous system disorders | lethargy | 38 | 3.11(2.26, 4.28) | 3.1(2.27, 4.24) | 1.63(1.18) | 3.1(2.37) |
| nervous system disorders | unresponsive to stimuli | 37 | 5.5(3.98, 7.6) | 5.49(4.01, 7.51) | 2.45(1.99) | 5.46(4.17) |
| nervous system disorders | dysarthria | 36 | 4.06(2.93, 5.64) | 4.05(2.9, 5.65) | 2.01(1.55) | 4.04(3.07) |
| respiratory, thoracic and mediastinal disorders | respiratory depression | 36 | 17.64(12.68, 24.55) | 17.59(12.61, 24.55) | 4.11(3.64) | 17.3(13.12) |
| infections and infestations | pneumonia aspiration | 35 | 3.75(2.69, 5.23) | 3.74(2.68, 5.22) | 1.9(1.43) | 3.73(2.83) |
| psychiatric disorders | nervousness | 34 | 3.01(2.15, 4.22) | 3(2.15, 4.19) | 1.58(1.1) | 3(2.26) |
| psychiatric disorders | depressed mood | 34 | 4.87(3.47, 6.82) | 4.86(3.48, 6.78) | 2.27(1.79) | 4.84(3.65) |
| injury, poisoning and procedural complications | head injury | 34 | 3.37(2.4, 4.72) | 3.36(2.41, 4.69) | 1.75(1.27) | 3.35(2.53) |
| psychiatric disorders | suicidal ideation | 33 | 6.01(4.27, 8.47) | 6(4.3, 8.37) | 2.58(2.09) | 5.97(4.48) |
| psychiatric disorders | aggression | 33 | 5.65(4.01, 7.95) | 5.63(4.03, 7.86) | 2.49(2) | 5.61(4.21) |
| psychiatric disorders | sleep disorder | 33 | 3.07(2.18, 4.33) | 3.07(2.2, 4.28) | 1.61(1.13) | 3.06(2.3) |
| eye disorders | miosis | 32 | 22.92(16.14, 32.56) | 22.86(16.06, 32.53) | 4.48(3.98) | 22.36(16.67) |
| injury, poisoning and procedural complications | product prescribing error | 29 | 4.84(3.36, 6.97) | 4.83(3.33, 7.01) | 2.27(1.75) | 4.81(3.54) |
| psychiatric disorders | abnormal behaviour | 28 | 5.17(3.57, 7.5) | 5.16(3.56, 7.49) | 2.36(1.83) | 5.14(3.77) |
| psychiatric disorders | restlessness | 24 | 3.57(2.39, 5.34) | 3.57(2.41, 5.28) | 1.83(1.26) | 3.56(2.54) |
| nervous system disorders | altered state of consciousness | 23 | 3.41(2.26, 5.13) | 3.4(2.25, 5.13) | 1.76(1.18) | 3.39(2.41) |
| nervous system disorders | hypokinesia | 22 | 6.16(4.05, 9.37) | 6.15(4.07, 9.28) | 2.61(2.02) | 6.12(4.31) |
| injury, poisoning and procedural complications | accidental overdose | 22 | 3.7(2.43, 5.63) | 3.7(2.45, 5.58) | 1.88(1.29) | 3.69(2.6) |
| psychiatric disorders | hallucination, visual | 21 | 3.32(2.16, 5.1) | 3.32(2.16, 5.11) | 1.73(1.12) | 3.31(2.31) |
| psychiatric disorders | nightmare | 21 | 3.98(2.59, 6.1) | 3.97(2.58, 6.11) | 1.98(1.38) | 3.96(2.76) |

ROR, reporting odds ratio; CI, confidence interval; PRR, proportional reporting ratio; IC, information component; EBGM, empirical Bayesian geometric mean; IC025, the lower limit of 95% CI of the IC; EBGM05, the lower limit of 95% CI of EBGM; PT: preferred term.
